# **Corrole Nanoparticles for Chemotherapy of Castration-Resistant Prostate Cancer and as Sonodynamic Agents for Pancreatic Cancer Treatment**

**DOI:** 10.1021/acs.jmedchem.2c01662

**Published:** 2022-12-14

**Authors:** Matan Soll, Vinay K. Sharma, Sally Khoury, Yehuda G. Assaraf, Zeev Gross

**Affiliations:** † Schulich Faculty of Chemistry, Technion − Israel Institute of Technology, Haifa 3200003, Israel; ‡ The Fred Wyszkowski Cancer Research Laboratory, Department of Biology, Technion-Israel Institute of Technology, Haifa 3200003, Israel

## Abstract

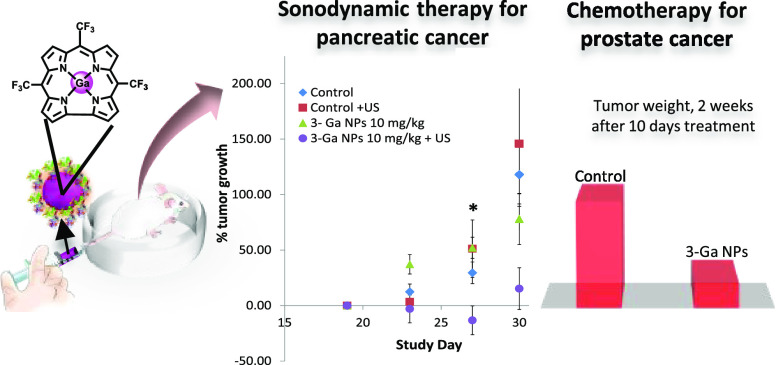

A nanoparticle-based system, composed of the gallium(III) complex of a minimally substituted corrole that is coated by transferrin as a targeting vehicle (**3-Ga** NPs), has been used for pre-clinical evaluation of its efficacy against human metastatic castration-resistant prostate cancer (mCRPC) tumor xenografts. All mice (*N* = 9) responded to a dose of 10 mg/kg, with a remarkable tumor growth inhibition of 400% following 2 weeks of treatment; Ames and hERG tests excluded potential concerns regarding mutagenicity and cardiotoxicity, respectively. Also demonstrated is the potential application of these **3-Ga** NPs as sonodynamic agents for the preclinical treatment of pancreatic cancer. 10 mg/kg **3-Ga** NPs combined with exposure to ultrasound waves (2 min of 1 MHz 0.1 w/cm^**2**^ twice a week) induced up to 77% tumor shrinkage. Consistently, tumor/tissue distribution and serum levels of **3-Ga** NPs in mice revealed high tumor specificity, favorable pharmacokinetics, fast absorption, slower redistribution, and very slow drug clearance.

## Introduction

The global prevalence and incidence of cancer is witnessing an increase due to the increase in the aging population.^1,2^ According to estimates from the United Nations “World Population Aging Report,” in 2019, 702 million people were aged over 65 years globally, and this figure is expected to reach 1.5 billion by 2050.^3^ Worldwide, prostate cancer is the second most frequent malignancy after lung cancer amounting 1.276 million new cases resulting in 358,000 deaths (3.8% of all cancer-related deaths in men) in 2018.^4^ Prostate cancer treatment heavily relies upon androgen deprivation therapy (ADT) via chemical castration, once this malignant tumor has spread outside the prostate.^5,6^ Secondary hormonal treatments such as abiraterone acetate or enzalutamide increase overall survival rates, though these hormonal agents are subject to the frequent emergence of intrinsic (primary) and acquired (secondary) anticancer drug resistance.^5,7^ Several promising androgen synthesis inhibitors, androgen receptor (AR) antagonists, and also heat shock protein modulators are under investigation as secondary hormonal treatment agents.^7^ Unfortunately however, most patients develop resistance to ADT, leading to castration-resistant prostate cancer (CRPC) within 18 to 36 months of treatment.^7^ Clinical oncologists rely on docetaxel as the first line treatment regimen for CRPC.^6^ Meanwhile, cabazitaxel serves as a second line treatment once the disease progresses to metastatic castration-resistant prostate cancer (mCRPC).^8^ To date, these treatment regimens improved slightly the life expectancy of prostate cancer patients.^8^ Therefore, mCRPC poses a formidable unmet need for novel and efficacious therapeutics, though a considerable extension of life expectancy could be reached in recent years.^9^

We have recently introduced the formulation of versatile protein-coated corrole nanoparticles (NPs).^10,11^ These novel NPs consist of the active drug and are coated with a targeting protein, without the need for loading an existing NP with the drug as in other drug delivery systems.^12,13^ In a most recent study, we have introduced the formulation of a much smaller molecular weight corrole 5,10,15-tris(trifluoromethyl)corrole and its Ga(III) complex (**3-Ga**)-based NPs coated with apo-transferrin (Tf).^14,15^ These **3-Ga** NPs displayed a potent efficacy *in vitro* toward a human CRPC cell line DU-145, with fast dynamics of receptor binding-dependent drug release and rapid induction of necrotic/apoptotic cell death. Upon **3-Ga** NPs binding to the cell surface, the **3-Ga** content of the NPs was immediately released and incorporated into the plasma membrane followed by redistribution into intracellular organelles. Moreover, within minutes of treatment, calcium influx, lysosomal destabilization, and reactive oxygen species (ROS) formation were apparent. Cumulatively, the mechanism of cell death was found to proceed via a necrotic/apoptotic pathway.^14^

In the present study, we demonstrate the therapeutic activity of Tf-coated **3-Ga** NPs against human CPRC tumor xenografts in mice. We show its initial safety and pharmacokinetics (PK) profile; the findings reveal that the NPs have a good safety profile, promising PK profile, and an efficacious therapeutic window *in vivo.***3-Ga** NPs also displayed great potential in a pancreatic human cancer cell line-based xenograft model with the use of sonodynamic therapy (SDT), revealing a promising tumor shrinkage of up to 77%. Overall, **3-Ga** NPs demonstrated a promising antitumor activity as a platform for the preclinical treatment of both CRPC and pancreatic cancer.

## Results

### Lead Compound Elucidation

**Figure 1:**
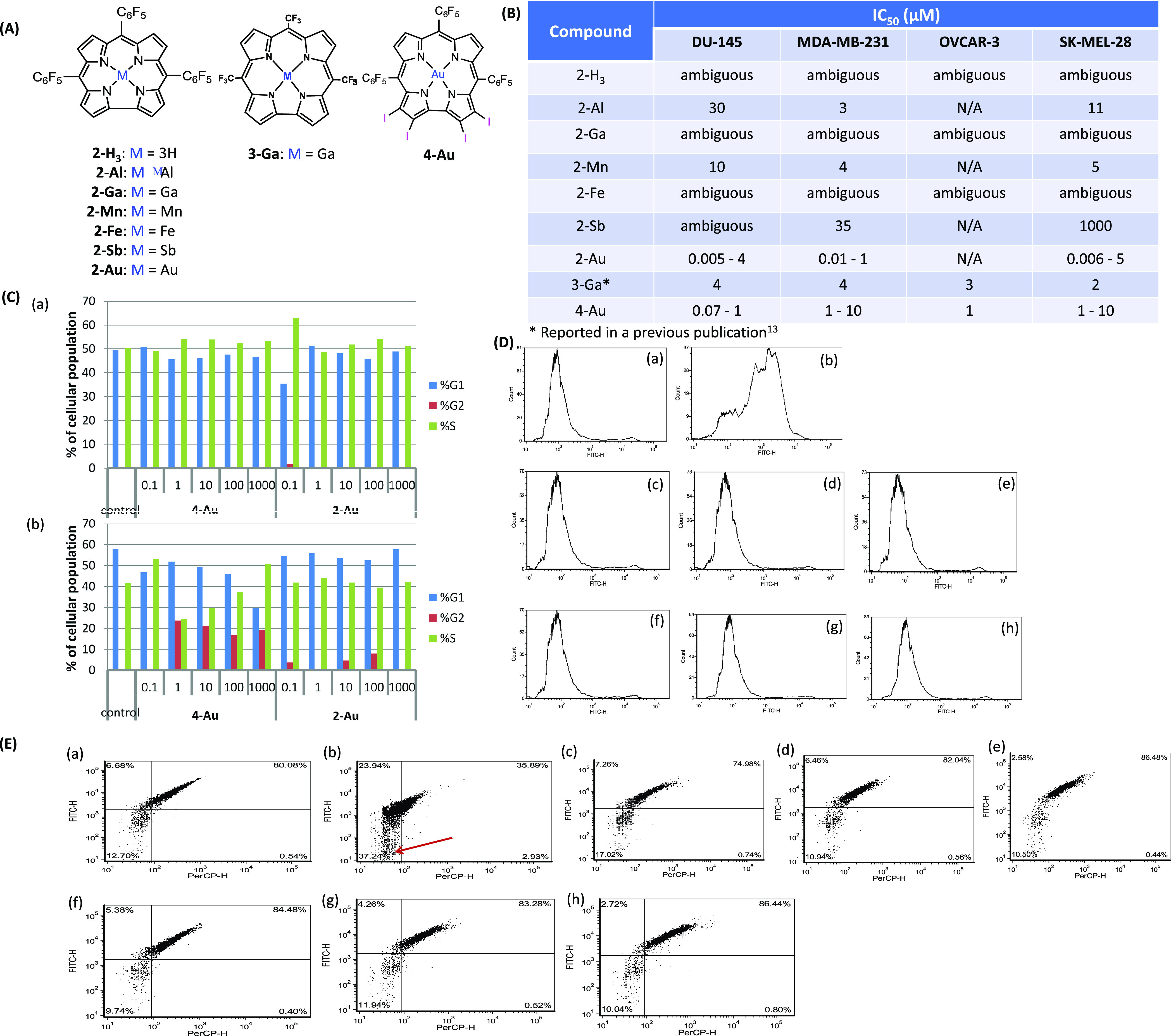
Selected examples from screening of possible corrole derivatives as preclinical antineoplastic agents: (A) structure of the investigated lead compounds and their metal complexes. (B) Table of IC_50_ values of different corrole derivatives and their Tf-coated NPs as assessed by a standard MTT assay to determine antitumor activity. (C) Cell cycle distribution of DU-145 cells as assessed by a flow cytomteric analysis assisted by a Vybrant DyeCycle Green Stain after treatment with increasing concentrations of **2-Au** NPs and **4-Au** NPs measured after (a) 24 h incubation and (b) 48 h incubation. (D) Flow cytometry analysis of DU-145 cells treated with an annexin V-FITC kit after 48 h incubation with: (a) Tf control; (b) positive control; (c–e) **2-Au** NPs 0.1, 1, and 10 μM, respectively; and (f–h) **4-Au** NPs 0.1, 1, and 10 μM, respectively. (E) Flow cytometry analysis of DU-145 cells treated with a DiBAC4(3) mitochondrial depolarization reporter kit after 48 h of incubation with: (a) Tf control; (b) carbonyl cyanide *m*-chlorophenyl hydrazone (CCCP positive control); (c–e) **2-Au** NPs 0.1, 1, and 10 μM, respectively; (f–h) **4-Au** NPs 0.1, 1, and 10 μM, respectively. Note the red arrow pointing toward the right quarter that indicates percentage of depolarized cellular population (obtained from CCCP-treated cells).

Multiple corrole derivatives and their NPs were tested on several cancer cell lines. These include several metal chelated derivatives of 5,10,15-tris(pentafluorophenyl)corrole (2-H_3_) (Figure 1A); β-substituted (iodinated and brominated) corroles and several of its metal chelates (only **4-Au** data are shown); different meso substituted corroles and their metal chelates (e.g., 5,15-di(pentafluorophenyl)-10-(imidazolyl)-corrole gallium(III), not presented); and the new synthetically available compound 5,10,15-tris(trifluoromethyl)corrole with different chelated metals (only the gallium(III) chelate **3-Ga** is presented in Figure 1A). Most of the corrole derivatives and their NPs did not display any substantial anti-neoplastic activity. The best candidates from the high molecular weight list of corrole derivatives were the gold complexes **2-Au** and **4-Au**. Both displayed submicromolar to single micromolar IC_50_ values in several human cancer cell lines (Figure 1C). **2-Au** was the most promising of the two with apparent values at single nanomolar values. However, these values were not consistent upon repeated experiments; investigation into the size of the formed NPs and their uptake by tumor cells did not eliminate the inconsistency of these results. Assessment of both apoptosis and mitochondrial depolarization did not reveal any active apoptosis 48 h after cell treatment with the corrole NPs (Figure 1D-E). However, there was an apparent elevation in the faction of cells residing in the G_2_ phase after treatment with **2-Au** and **4-Au** NPs, more so with the latter (Figure 1B). Though there was an apparent effect on the cell cycle progression of the treated cells, there was no apparent cell death outcome accompanying the cell cycle arrest. Furthermore, the cell population within the culture wells appeared to be dividing freely and completely covering the wells’ surface (data not shown).

In contrast to the larger corroles, **3-Ga** displayed a consistent activity toward several cancer cell lines: in some of which the anticancer effect was immediate (4 h in the DU-145 cell line), whereas in other tumor cell lines the antitumor effect was only apparent after 48 h of incubation.^14^**3-Ga** NPs displayed active apoptosis in conjunction with necrosis as revealed by several assays (described in detail in our recent publication^14^), hence being thus far, the first and only corrole to display such activity. The results suggest that the mechanism of cell death is via the necroptosis pathway.^14^ In-depth investigations into the activity of **3-Ga** revealed that it bears various biological effects, depending on the cell type and tissue origins.^14^ The efficacy of **3-Ga** was most impressive in the CRPC cell line DU-145. We have hence focused our primary efforts on prostate cancer as the lead indication for **3-Ga** NPs, by proceeding with the assessment of its pharmacokinetics, safety, and *in vivo* efficacy.

#### Pharmacokinetics (PK)

We have previously shown that corrole NPs could be readily formulated under ambient conditions directly in aqueous conditions with albumin as a coating protein.^10,11,14^ In the present study, we have chosen Tf to serve as both the coating protein and the targeting ligand that might mediate the specificity of the NPs toward CPRC cells. High expression of the transferrin receptor (TfR) is typically found in rapidly proliferating cells, including various types of cancer.^16^ This allows the use of Tf as a targeting moiety toward malignant tumors and has been identified as a good target for various NP delivery systems.^17−20^

The mode of administration chosen for **3-Ga** NPs was intravenous (IV). This is preferred due to the nature of the NPs, i.e., their size (50 nm) and the lack of reasonable drug release/redistribution when there is no binding to a receptor on the cell surface.^14^ Furthermore, IV administration is also preferred in the clinical setting. **3-Ga** NPs were administered *in vivo* at two doses to obtain the PK profiles of the compounds and accordingly to determine if the PK behavior was linear by comparing the values of the area under the curve (AUC).

**Figure 2:**
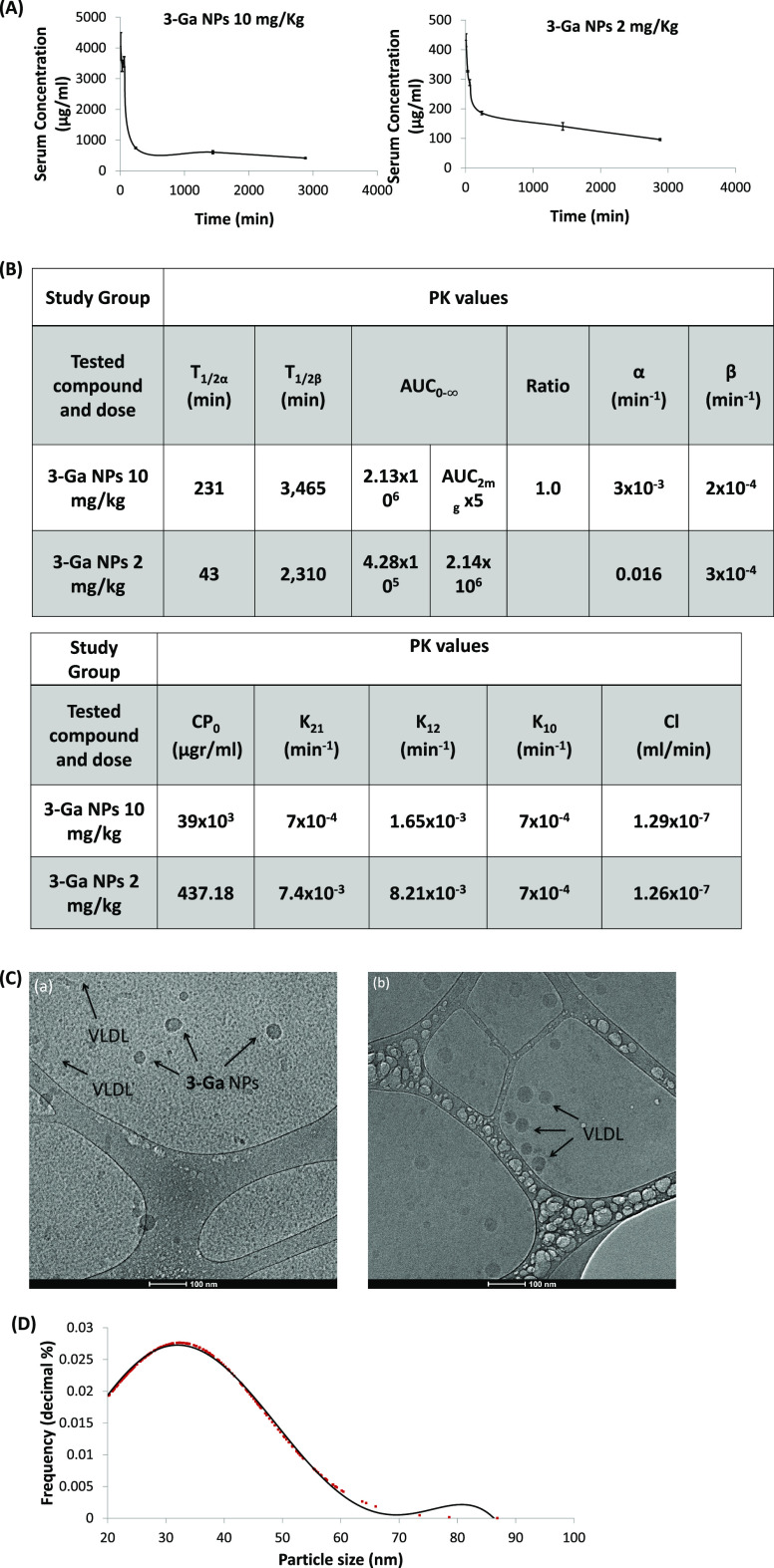
Assessment of **3-Ga** NPs pharmacokinetics (PK) upon IV administration: (A) PK profiles of **3-Ga** NPs at doses of 10 and 2 mg/kg; (B) table of the derived PK parameters to each dosage regime; (C) Representative cryo-TEM images of sera derived from mice, 30 min after treatment with (a) **3-Ga** NPs and (b) vehicle; and (D) distribution analysis of NP size determined from 12 separate fields in three distinct samples of **3-Ga** NPs treated mice.

The PK profile of **3-Ga** NPs may be best described by a two-compartment model and fast redistribution with a relatively short half-life (Figure 2A) followed by slow elimination with a half-life of 38–58 h, depending on the dose. **3-Ga** NPs displayed a nearly linear behavior at the tested doses (Figure 2B). Values of K12 reveal a fast absorption of **3-Ga** with slower redistribution into the central compartment (K21) and a very slow clearance of the compound (Cl, K10) (Figure 2B). In terms of PK parameters, **3-Ga** NPs display favorable attributes for therapeutic use in the clinical setting.

Thirty minutes after IV administration of **3-Ga** NPs in mice, serum samples were derived and prepared for Cryo-TEM imaging as well as for evaluation of particle integrity (Figure 2Ca). Individual particles could be detected and differentiated (by the virtue of contrast and geometry alone) from lipoproteins (depicted by arrows), displaying similar sizes (Figure 2Cb). The estimated diameter of **3-Ga** NPs uncovered a smaller size (32.4 ± 14.4 nm) relative to the same NPs in PBS prior to IV administration (∼50 nm).^14^ Thus, prior estimations of NP integrity in serum/circulation proved correct,^9^ though with some changes in the overall size of the NPs. The change in particle size could be attributed, at least in part, to **3-Ga** redistribution into peripheral tissue or to hemodynamic forces.

#### Therapeutic Efficacy of **3-Ga** NPs in Human CPRC Tumor Xenografts in Mice

**Scheme 1:**
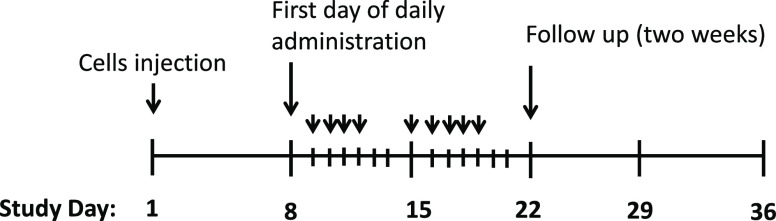
Schedule of NP Injection in the *In Vivo* Model of Prostate Cancer Mice received initial IV administration of test components on day 8 followed by IV administration of treatments on days 9, 10, 11, 12, 15, 16, 17, 18, and 19 (depicted by black arrows).

The human prostate cancer cell line DU-145, which was isolated from brain metastasis, is considered one of the first cell lines to model CRPC with differential abilities to metastasize to other organs *in vivo*, depending on the murine model used.^21^ We decided to use the male nude mouse model (Foxn1^nu^) since a subcutaneous injection of DU-145 cells (which displayed a strong susceptibility toward **3-Ga** NPs in previous studies) produced tumors with a robust phenotype.^22^ Upon tumor appearance (tumors reached a mean volume of ∼90 mm^3^), mice were divided into three groups according to similar tumor sizes, and treatments were initiated with **3-Ga** NPs for 2 weeks, as outlined in Table 1. After treatment, mice were monitored for two additional weeks for body weight and tumor size. The study timeline schedule is depicted in Scheme 1.

**Table 1:** Group Assignment and Treatment

test Item	test item stock solution for IV injection	ROA^a^	dose volume
vehicle	100% PBS + Tf (0.2 mg/mL)	IV	5 mL/kg
**3-Ga** NPs 5 mg/kg	1 mg/mL	IV	
**3-Ga** NPs 10 mg/kg	2 mg/mL	IV	

a ROA: route of administration.

**Figure 3:**
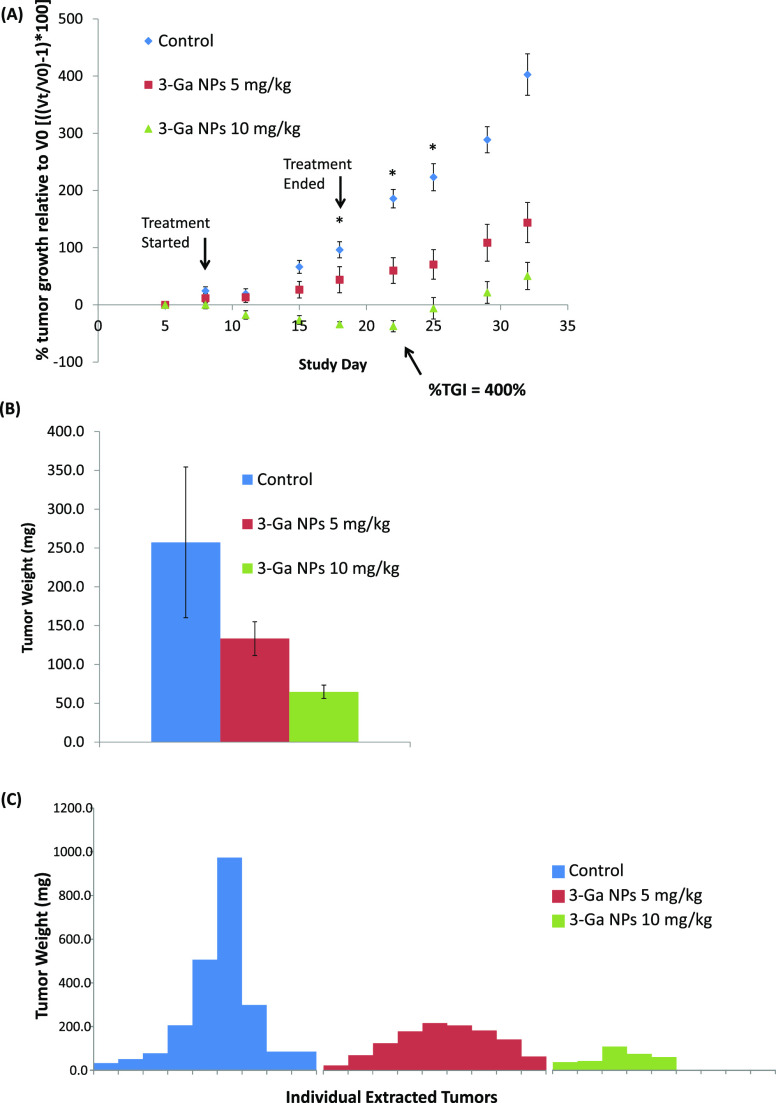
(A) Xenograft model of nude mice (Foxn1^nu^) harboring human DU-145 hormone refractory CRPC tumors. Tumors were measured for width (*W*) and length (*L*) using a caliper and volume (*V*) was calculated according to the following equation: *V* = *LW*^2^/2. Data were then normalized (relative to initial tumor size at day 5) and plotted. Results represent mean values ± SEM of all mice in each group (*n* = 9, *p < 0.008 at **3-Ga** NPs 10 mg/kg vs control, according to *t*-test). (B) Mean tumor weight at end point. (C) Individual tumor weight distribution at end point.

The NPs displayed very good efficacy toward human DU-145 CRPC tumors studied in two distinct experiments (Figure 3, Figures S2 and S4). Inhibition of tumor growth was readily apparent in a dose-dependent manner both at 5 and 10 mg/kg, hence achieving statistical significance from day 19 to 25 at a dose of 10 mg/kg (Figure 2A). Treatment with 10 mg/kg achieved up to 60% tumor shrinkage relative to the initial tumor volume with an outstanding TGI value of 400% (Figure 3A, day 22). The high variability in the control group (Figure 3C) rendered the statistical significance both in the 5 and the 10 mg/kg (days 25 to 36) slightly weaker. In contrast, in a former experiment at a dose of 5 mg/kg, this treatment displayed a marked TGI with statistical significance throughout the entire experiment (Figure S2). The remarkable therapeutic efficacy is also apparent from the very small variability in the treatment groups relative to the control groups, implying a consistent TGI. That is, all treated mice displayed a robust marked TGI (Figure 3C). On day 18, the last day of treatment, mice started to show weight loss that continued until day 23 with a recovery of weight and overall health within a week (Figure S3). For ethical considerations, we had to sacrifice four mice due to low weight; no mortality of mice was observed. Substantial tumor shrinkage could be observed within the first week of treatment and there were no apparent side effects. Untoward toxicity appeared only in the second week of treatment, implying that an intermittent regimen or an alternative treatment schedule may lead to good efficacy for optimal treatment of CRPC without the untoward toxicity that appeared after the second week of treatment. The recovery of mice from weight loss implies that this toxic side effect was transient and reversible and may not have deleterious long-term effects on animal health. The 10 mg/kg treatment dose was obviously excessive given the known PK of the NPs, possibly leading to an extremely high dose of the NPs in the serum. The slow clearance of NPs implies that a single high dose once a week or even every other 2 weeks might be sufficient to achieve antitumor efficacy in the absence of apparent toxicity. In several mice, a necrotic epidermis at the site of injection could be detected after 2 weeks of treatment. This side effect was also ephemeral, with the recovery of the tail skin within a week.

Upon histological examination (H&E staining) of tumors, the inflammation score was low in the control group (Table S1). **3-Ga** NP 5 and 10 mg/kg treated groups displayed a high mean inflammation score response of 2.14 and 2.6, respectively. In **3-Ga** NP 5 and 10 mg/kg treated groups, a clear peripheral infiltration of macrophages and lymphocytes could be detected (Figure S5). Cell differentiation was quite constant with a low grade of neutrophils and a relatively moderate and identical percentage of macrophages and lymphocytes within the tumor. This active peripheral infiltration of macrophages and lymphocytes into the tumor in the treated groups is very encouraging and might indicate the recruitment of the immune system into the tumor aided by the action of **3-Ga.**

In a former experiment (Figure S2), in which the maximal dose was 5 mg/kg, there was a tumor shrinkage of up to 20% relative to the initial volume of the tumors (day 17) with statistical significance throughout the experiment and without any apparent side effects (Figure S2). The reproducibility of the 5 mg/kg regimen efficacy in the same model substantiates the potential of this treatment to be implemented in the clinical setting, and hence future efforts directed at the optimization of the treatment regimen are warranted.

These impressive results prompted us to investigate whether **3-Ga** NPs could have an antitumor activity on pancreatic cancer as well. Pancreatic cancer is designated as a rare disease, with an annual incidence of 12.9 cases per 100,000.^23,24^ Nonetheless, the annual death rate is 11 deaths per 100,000 due to the fact that pancreatic cancer patients have a very dismal prognosis with a 5 year survival rate of 5%. Although the incidence of pancreatic cancer is significantly low, it is the third most common cause of cancer-related deaths in the USA.

**Figure 4:**
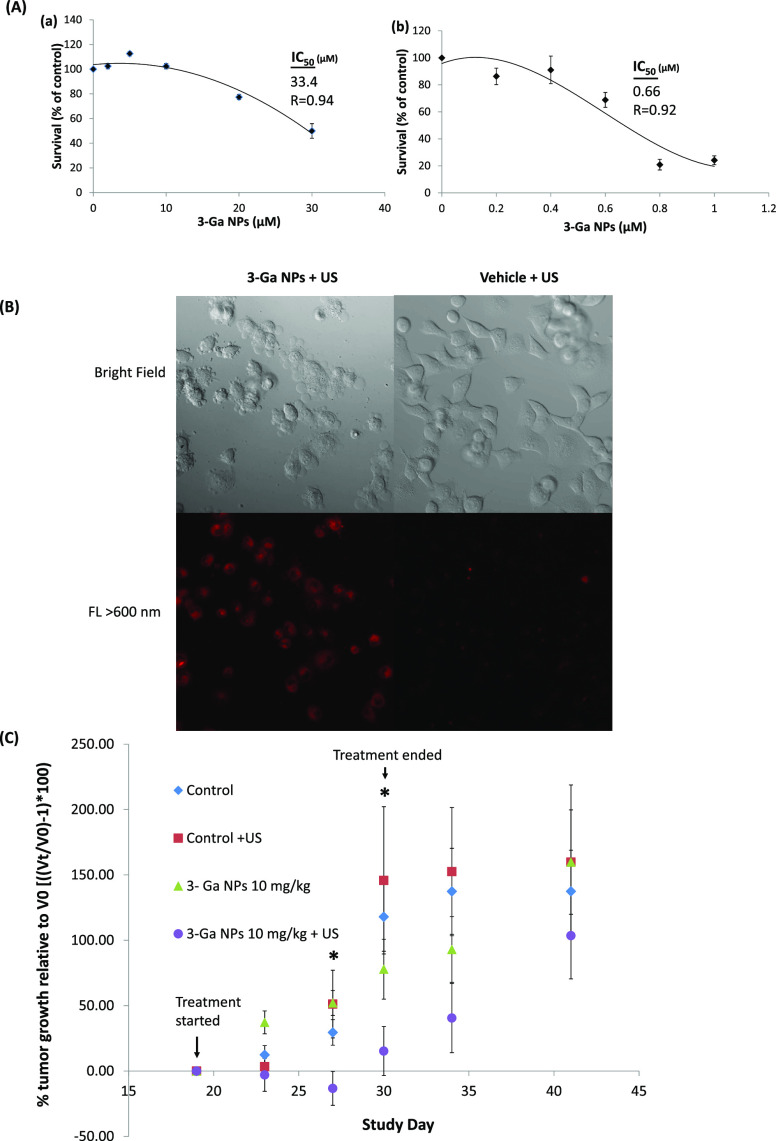
Efficacy of **3-Ga** NPs against pancreatic cancer cells *in vitro* and *in vivo* in tumor xenografts in mice: (A) IC_50_ values as assessed by the MTT assay using Panc-1 cells treated with (a) **3-Ga** NPs and (b) **3-Ga** NPs + US 0.1 w/cm^2^ 1 MHz for 30 s. Data points are mean values ± SEM of 3 independent experiments. (B) Confocal microscopy of Panc-1 cells incubated for 30 min with 1 μM **3-Ga** NPs (red fluorescence) and exposed to US 1 MHz 0.1 w/cm^2^ for 30 s, relative to cells incubated with vehicle for 30 min and exposed to US 1 MHz 0.1 w/cm^2^ for 30 s. Fluorescence was recorded using a 40× objective and an LSM700 confocal microscope supported with Zen software. Samples were excited at 405 nm (3%) for **3-Ga** detection. Representative images of 12 separate fields and three independent experiments. (C) Xenograft model in nude mice (Foxn1 nu) implanted with Panc-1 pancreatic cancer cell tumors. The width (*W*) and length (*L*) of the tumors were measured using a caliper, and the tumor volume (*V*) was calculated according to the following equation: *V* = *LW*^2^/2; data were normalized (i.e., relative to initial tumor size at day 5) and plotted. Results represent mean values ± SEM of all mice in each group (*n* = 7, *p < 0.05 at **3-Ga** NPs 10 mg/kg + US vs control + US, according to *t*-test).

Though **3-Ga** NPs displayed a robust efficacy both *in vitro* and *in vivo* toward prostate cancer cells, their *in vitro* antitumor activity toward a pancreatic cancer cell line was much more limited (*vide infra*, Figure 4A). Though the innate biological effect of **3-Ga** NPS was insufficient for the induction of cell death in pancreatic cancer, previous investigations on corroles and their use as sonodynamic therapy (SDT) agents prompted us to assess whether these novel NPs could be used as SDT agents for the treatment of pancreatic cancer.

#### Sonodynamic Therapy (SDT) Treatment Efficacy against Pancreatic Cancer

We have recently reported the potential of corroles to be used as sonosensitizers for SDT.^25^ Corroles and their formulated NPs were found to initiate the formation of singlet oxygen radicals upon sonication in aqueous conditions. Thus, we have conducted initial *in vitro* experiments to ascertain whether **3-Ga** NPs could be used as sonosensitizers in a human pancreatic cancer cell line model. Indeed, **3-Ga** NPs displayed a strong cytotoxic activity after ultrasound (US) exposure, inducing cell death with a 51-fold lower IC_50_ values (0.66 μM) than without exposure to US waves (33.4 μM, Figure 4A). Cell apoptosis represented by plasma membrane blebbing could be detected upon US exposure and treatment with 1 μM **3-Ga** NPs, whereas no such membrane blebbing occurred in drug-free control cells solely treated with US (Figure 4B). These remarkable *in vitro* results prompted us to test the activity of **3-Ga** NPs along with US exposure *in vivo* against pancreatic cancer. The *in vivo* experiment using a human Panc-1 pancreatic cancer cell line xenograft model revealed a substantial TGI, under co-treatment with **3-Ga** NPs and US, relative to US exposure alone (Figure S6). This initial experiment included daily administration of 5 mg/kg **3-Ga** NPs and daily exposure to US, 5 days a week for 2 weeks.

**Scheme 2:**
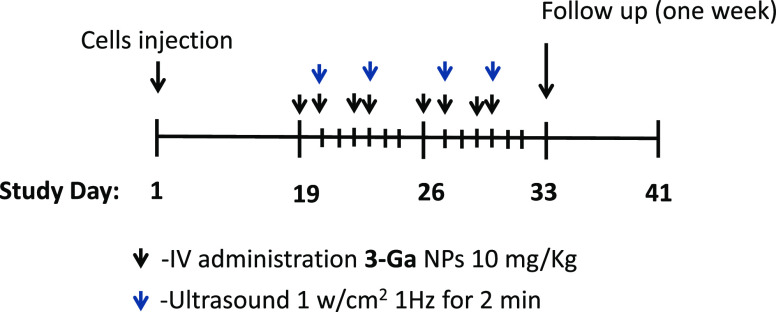
Treatment Timeline Schedule of the Pancreatic Cancer *In Vivo* Model Mice received an initial IV administration of the test items on day 19 followed by IV administrations on days 20, 22, 23, 26, 27, 29, and 30, depicted by black arrows. US treatment commenced on day 20 followed by repeated treatments on days 23, 27, and 30, depicted by the upper dark blue arrows.

However, the above-described protocol led to some untoward side effects and formation of skin scaring, toughening, and burns at the site of the US transducer contact over a long period of time. Therefore, we used a different treatment regimen in our main experiment: introducing US waves only twice a week for 2 weeks with a 2–3 day interval between sessions. When tumors reached a mean volume of ∼90 mm^3^, mice were stratified into three groups according to tumor size; mice with a similar mean tumor volume were treated with Tf-coated **3-Ga** NPs and US for 2 weeks, as shown in Table 2. Following treatment, mice were monitored for an additional week for body weight and tumor size. The treatment schedule is depicted in Scheme 2, which outlines the control experiments and the three doses of **3-Ga** NPs that were used.

**Table 2:** Group Assignment and Treatment

test item	test item stock solution for injection	ROA^a^	dose volume
vehicle	100% PBS + Tf 0.2 mg/mL	IV	5 mL/kg
vehicle + US	100% PBS + Tf 0.2 mg/mL	IV	
**3-Ga** NPs 1 mg/kg	0.2 mg/mL	IV	
**3-Ga** NPs 1 mg/kg + US	0.2 mg/mL	IV	
**3-Ga** NPs 5 mg/kg	1 mg/mL	IV	
**3-Ga** NPs 5 mg/kg + US	1 mg/mL	IV	
**3-Ga** NPs 10 mg/kg	2 mg/mL	IV	
**3-Ga** NPs 10 mg/kg + US	2 mg/mL	IV	

a ROA: route of administration.

The results obtained in this small experiment (*N* = 3) with a dose of 5 mg/kg **3-Ga** NPs + US were disappointing (Figure S7B), likely because we have substantially reduced the exposure of tumors to US waves from 5 to 2 days a week. Obviously, the 1 mg/kg treatment regimen did not display any substantial results as well (Figure S7A). However, very good results were achieved with a dose of 10 mg/kg **3-Ga** NPs + US, which now attained statistical significance (Figure 4C). A detailed examination of the results obtained on day 27 (the end of US treatment) revealed that 5 of the 7 mice displayed tumor shrinkage relative to the initial tumor volume: −0.67, −2.95, −3.79, −32.95, −42.16%, and a staggering −78.36%. The remaining two mice displayed a mild 20–25% tumor volume increase, in comparison with the 40–200% tumor volume increment in control tumors (both with and without US). This relative advantage continued up to day 30 of the treatment (last day of treatment): two mice displayed relative tumor shrinkage of −34.4% and −77.15%, whereas the remaining mice displayed mild increases in tumor volume of 9.38% up to 38.34%, relative to 153.6–188.2% increases in tumor volume in the controls (with and without US).

It is still important to point out that the advantage of the **3-Ga** NP 10 mg/kg + US treatment did not last too long. Once the treatment was completed, most tumors regained their growth to levels only slightly lower than their control counterparts. Only in 2 out of the 7 mice, the tumors remained without a change in their shrunken form (−33% and −77%). It appears that the treatment has made some of these tumors rather dormant, although this phenomenon did not attain statistical significance. These treatment regimens did not display any form of side effects and there was no loss of body weight in any of the treated groups (Figure S7C). These findings are in accord with our previous assumptions regarding the successful treatment of prostate cancer tumor xenografts where optimization of the treatment could lead to better results without side effects.

#### Uptake and Specificity of **3-Ga** NPs into Tumors

In our recently published *in vitro* study, **3-Ga** NPs displayed high specificity of drug-payload release toward prostate cancer cells; this only occurred following binding to the plasma membrane.^14^ This drug release could be competitively inhibited by the free ligand, hence blocking the NPs from binding to the tumor cell surface.

**Figure 5:**
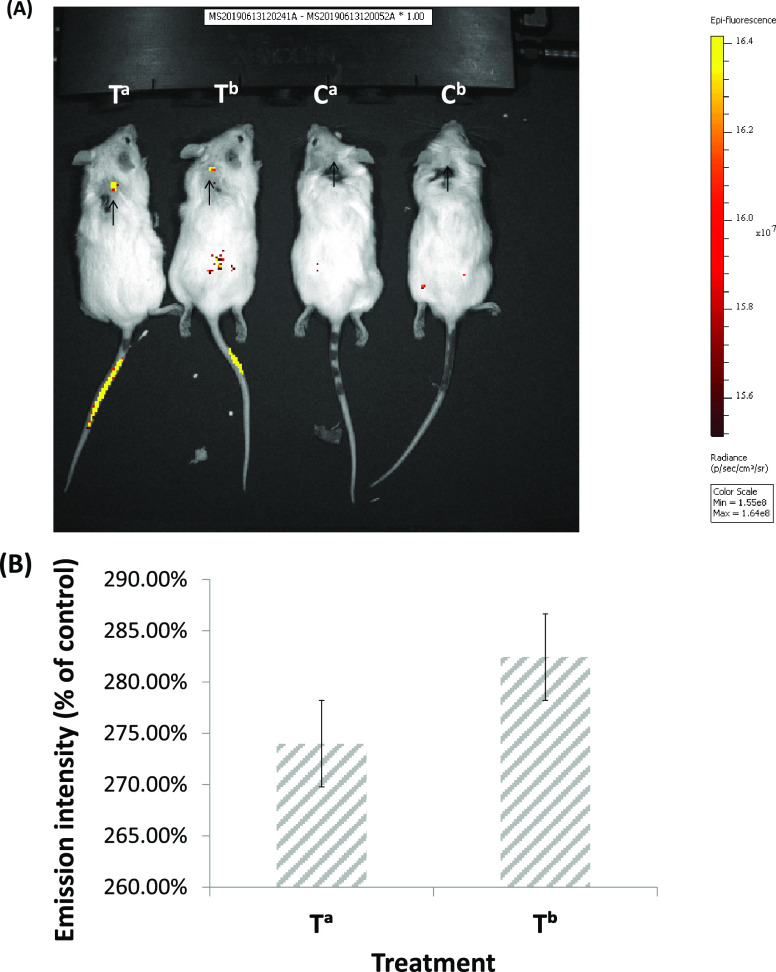
Tumor targeting specificity of **3-Ga** NPs. (A) NSG mice bearing human Panc-1 tumors (65 mm^3^), which received a single IV injection of either **3-Ga** NPs (Ta and Tb, 5 mg/kg) or vehicle (Ca and Cb, ∼120 μL), were imaged at 2.5 h post-injection using a noninvasive small whole-body fluorescence imaging system; shown are representative images from two distinct experiments with 3 mice in each group; (B) quantitative analysis of emission of tumor area (depicted by arrows, region of interest [ROI]) relative to corresponding ROI in the control mice. Results represent mean ± SEM of all mice in each group (*n* = 3).

The high specificity and uptake of **3-Ga** NPs are particularly important in the assessment of the efficacy in the US-aided xenograft model of pancreatic cancer. This is because the applied US apparatus has no focusing ability and there is very little control of the homogeneity of US waves that are emitted through the subcutaneous tumor. Therefore, it was imperative to assess the specificity of **3-Ga** uptake into the tumor. IV administration of 5 mg/kg **3-Ga** NPs to NSG mice bearing human pancreatic cancer Panc-1 tumor xenografts revealed a substantial uptake of **3-Ga** into the tumors and in a specific manner, as soon as 2.5 h after administration (Figure 5, Figure S8). Apart from the obvious fluorescence emission from the site of injection at the tail, strong emission could be detected from the exact site of the Panc-1 tumor site at the back neck of the treated mice (T^a^ and T^b^). No such emission could be detected at the tumor site of mice treated with the vehicle (C^a^ and C^b^). Remarkably, the levels of emission from the tumor correspond to the levels detected at the site of injection in the tail.

#### Preliminary Safety Studies upon Injection of **3-Ga**

Assuming that the treatment of patients in the clinical setting would include the use of **3-Ga** in a form of NPs, given one of many possible identities of the coating protein, we decided to assess the safety of unformulated **3-Ga** in preliminary *in vitro* assays. The premise is that NPs would show very little toxicity since preliminary data suggested that significant drug cargo release from NPs requires binding of the coating protein to the cell surface via specific interaction with the appropriate receptor.^14^ Therefore, to assess the toxicity of **3-Ga** as a new molecular entity, one must study it as is, i.e., without any formulation that leads to its assembly as protein-coated NPs.

#### Mutagenicity Ames Test

Despite the development of tests to determine the mutagenicity of compounds via DNA damage, the Ames test retained its primary role in the testing of chemicals for their mutagenicity and safety for commercial and medical uses.^26^ Briefly, the Ames test uses 5 bacterial strains (*Salmonella* and *E. coli*) that are exposed to a given compound with and without an enzymatic bioactivation system derived from rodent liver (S9). Each bacterial strain has a different loss of function mutation in a gene essential for the biosynthesis of a required amino acid, histidine in *Salmonella* or tryptophan in *E. coli*, such that they cannot grow and form colonies on agar plates lacking these amino acids. If exposure to the tested compound reverses this dependency on the above amino acids, it is obvious that a mutation has occurred, which according to the number of colonies formed implies mutagenicity of the tested compound.

**Figure 6:**
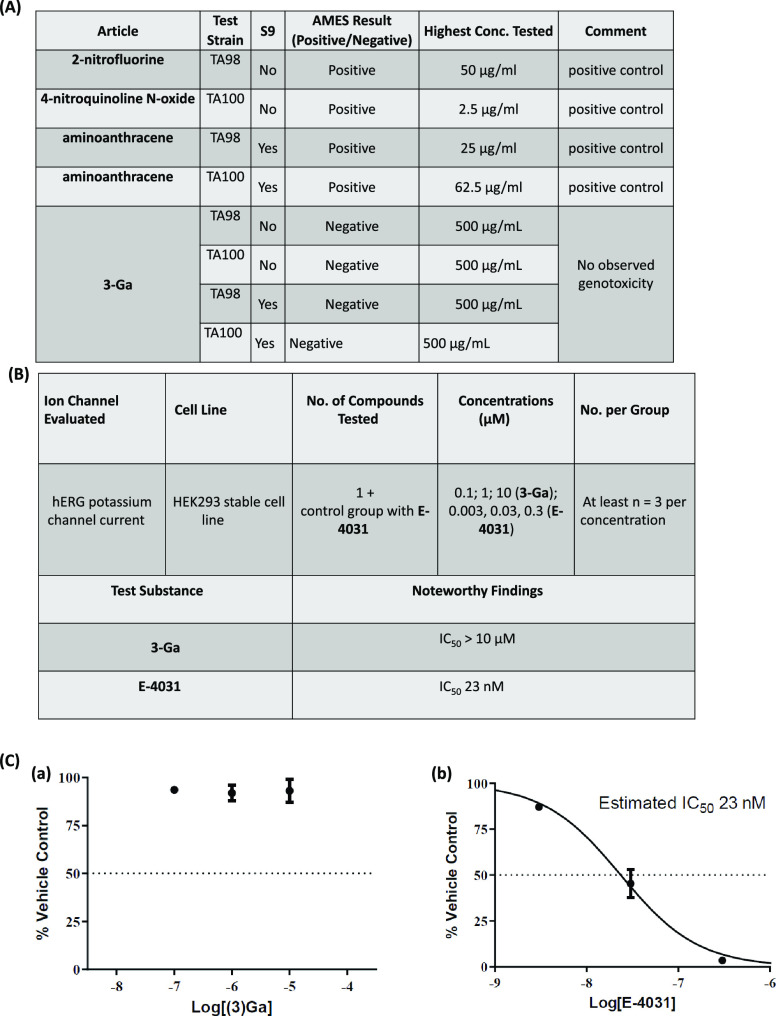
Preliminary safety profile of **3-Ga**: (A) Summary of Ames test end point results; the tested concentrations of **3-Ga** were as follows: 15, 31, 62, 125, 250, and 500 μM. (B) hERG test summary and results; tested concentrations of **3-Ga** were as follows: 0.1, 1, and 10 μM. (C) Dose–response relationship for (a) **3-Ga** on hERG tail current; (b) E-4031 on hERG tail current. Data shown are mean values ± SEM (*n* = 3).

Assessing the safety of **3-Ga** in two *Salmonella* strains TA98 and TA100 revealed no genotoxicity, although at the highest concentration, some cytotoxicity was apparent (Figure 6A, Figure S9). No increase in colony formation could be observed relative to the baseline (Figure S9). Positive controls displayed a more than 3-fold increase in colony formation relative to the baseline as previously reported.^27^

#### hERG Test

The human ether-a-go-go related gene (hERG) encodes for the inward rectifying voltage-gated potassium channel in the heart (IKr), which is involved in cardiac repolarization.^28^ Inhibition of the hERG current causes QT interval prolongation, resulting in potentially fatal ventricular tachyarrhythmia called Torsade de Pointes. HEK293 cells stably transfected with hERG are used to assess the effect of different substances at increasing concentrations (typically, 0.1, 1, and 10 μM). Using the QPatch HTX (Sophion Bioscience A/S) apparatus, pre-programmed voltage-clamp steps are applied and electrophysiological recording is performed upon exposure to the tested compound. DMSO (0.25–0.3%) was used as a negative control and E-4031, a known effector and toxin, as a positive control. Effects on tail current were plotted against given concentrations, and the estimated IC_50_ values were calculated (Figure 6C). When compared to vehicle treatment, **3-Ga** produced a decrease in hERG tail currents, which reached 6.98% inhibition at the highest concentration tested (10 μM, Figure 6Ca, Figure S10A,B). The compound IC_50_ value could not be calculated, and therefore, it is estimated to be greater than 10 μM, a value which is considered safe by standard commercial requirements (Figure 6B). The reference standard E-4031 was tested in a dose–response curve, and its inhibitory effect was consistent with published data (Figure 6Cb, Figure S10C,D). The control consisting of 0.2% DMSO (used as vehicle for **3-Ga** solubility) did not affect tail current at all, while the addition of E-4031 to the same test wells almost completely inhibited hERG tail current (Figure S10E,F).

## Discussion

**3-Ga** NPs exhibited very promising *in vivo* results, with a remarkable efficacy toward CRPC in a dose-dependent manner. The untoward toxicity associated with **3-Ga** NPs at 10 mg/kg was only apparent in the middle of the second week of the treatment regimen. Besides the apparent weight loss, there were signs of necrosis upon the skin of the tail where the NPs have been injected. The maximal tolerated dose (MTD) therefore would be 10 mg/kg using this treatment regimen. Tumor shrinkage was substantially apparent from the first few days of treatment followed by a continuous tumor shrinkage until the last days of **3-Ga** NP administration. Within the first few days of the second week of **3-Ga** NPs administration, the treatment has reached its maximal therapeutic impact and started to induce adverse effects and weight loss. These toxic side effects were not detrimental, though the substantial weight loss requires the inevitable sacrifice of four mice due to ethical considerations, but the remaining five mice recovered and regained weights that are consistent with the control group. The 5 mg/kg regimen appears to be an effective dose, although it leads to much less TGI and tumor regression based on the two independent **3-Ga** NPs treatment efficacy experiments (*n* = 7, *n* = 9).

The treatment regimen was excessive given the known PK of the formulation. This allowed us to assess the therapeutic window of such a treatment and its side effects. Given the slow clearance of the NPs, it appears that Tf-coated **3-Ga** NPs allow a very broad range of serum concentrations before the onset of side effects. Therefore, more efficacious therapeutic regimens could be devised and tested, leading to enhanced antitumor activity and maintenance of tumor shrinkage throughout several weeks. Future efforts in optimizing the treatment regimen could lead to the substantiation of **3-Ga** NPs as a potential treatment modality for CRPC in clinical setting. Initial promising results from known industrial pharmacovigilance assays including the hERG assay and the Ames test support the relative safety of this treatment modality.

In the current study, we have also revealed the potential of **3-Ga** NPs as a sonosensitizer agent for SDT. Pancreatic cancer is a leading cause of cancer-related deaths in the Western world with very limited therapeutic options and bleak survival; the overall 5 year survival rates currently stand at disheartening 9%.^29^ Apparently, **3-Ga** NPs did not display any therapeutic effect as monotherapy on the pancreatic cancer-derived cell line Panc-1, both *in vitro* and *in vivo*. However, the combination of NP treatment with US allowed a beneficial therapeutic effect that led to substantial TGI and regression. These therapeutic effects were not long-lasting, and most of the tumors resumed their growth after cessation of treatment. Still two of the seven treated mice displayed tumor regression that reflected a complete inhibition of tumor growth in the two weeks following the completion of treatment. Fortunately, the treatment regimen did not lead to untoward side effects or weight loss as displayed by the regimen tested in the CRPC *in vivo* model using **3-Ga** NPs. Note that the above beneficial therapeutic effects were achieved using a simple transducer with relatively unfocused, non-homogeneous, and low-energy US waves (transducer used in the clinic for orthopedic applications) and in a subcutaneous heterotopic tumor xenograft model. Obviously, the pancreas is located in a much deeper visceral location; therefore, a focused US apparatus that is optimized for **3-Ga** activation should be devised. The acoustic availability of the pancreas and therefore of the pancreatic cancer tissue was demonstrated using high intensity focused ultrasound (HIFU).^30^ The beneficial effects of HIFU *per se* in pancreatic cancer patients have also been demonstrated with palliative effects in which a substantial tumor-related pain reduction was achieved in most patients after HIFU treatment.^31^ The combination of HIFU optimized for **3-Ga** activation and **3-Ga** NPs may prove to be effective in future treatment of pancreatic cancer.

The specific uptake of **3-Ga** by the tumors was demonstrated as well, supporting our previous hypothesis from early *in vitro* studies.^14^ Regarding pancreatic cancer, data suggest that genetic alterations in this malignancy affect both collagen architecture and signaling pathways, resulting in increased fibrosis and tissue tension.^32^ Therefore, the use of NPs might not be the therapy of choice in the clinical setting due to poor pancreatic tumor penetration. This may be the case when using monotherapy; however, the combination with US-dependent activation of **3-Ga** may lead to local alleviation of tissue tension and the formation of a more permeable environment that allows the uptake of NPs by the tumor and *in situ* activation of **3-Ga**. Future investigations in an orthotopic pancreatic cancer model and a **3-Ga**-based HIFU treatment could determine whether or not this assumption is valid.

In the current research, we have also put emphasis on optimizing the formulation of **3-Ga** NPs regarding the treatment efficacy in xenograft mouse models *in vivo.* We also examined the purity of synthesized **3-Ga**^33^ and the reproducibility/homogeneity of the formulated NPs (Figure S11). We have also provided a protocol yielding robust size and homogeneity of NPs with very low polydispersity of around 0.0593. These are highly homogenous NPs, as may be appreciated from the cryo-TEM images of the NPs in PBS (Figure S11C).

Collectively, **3-Ga** NPs emerge as a promising nanomedicine modality for the treatment of two distinct cancers, while other tumors are awaiting future evaluation. These efforts may put emphasis regarding which type of coating protein could be the most efficacious as it serves not only as an encapsulation protein but also as a tumor-targeting ligand. The simple formulation allows the introduction of any given protein in ambient conditions that will allow maintenance of proper structural protein features as well as efficient **3-Ga** encapsulation. Different coating proteins may serve to target other cancers like in the case of heregulin-modified protein directed at the human epidermal growth factor receptor (HER), which in combination of the gallium complex of a sulfonated corrole was proven very effective in the case of triple negative breast cancer.^34^ The current paradigm of precision medicine could be achieved with relative ease, considering the simplicity of the formulation and encapsulation-targeting ligand. Given the genetic background and companion diagnostics of the patient and the tumor, one could devise the optimal coating protein for the best outcome using protein coated **3-Ga** NPs. This rather facile and relatively inexpensive technological advancement may allow affordable future use of these NPs in the clinic.

## Conclusions

**3-Ga** NPs proved to be remarkably efficacious toward CRPC *in vitro and in vivo*, leading to an impressive tumor regression. All tumor xenograft bearing mice displayed a therapeutic response to the treatment regimen already within the first few days of treatment. During the first week, no side effects or toxic events were recorded. In contrast, the second week of treatment revealed signs of untoward toxicity observed at the end of the treatment regimen, leading to the conclusion that daily administration of 10 mg/kg for 2 weeks is not the ideal treatment regimen and that a more intermittent regimen might display better therapeutic outcomes, while being free of side effects, toward the establishment of an optimal treatment regimen.

The preliminary assessment of **3-Ga** NPs as SDT agents revealed very promising results given the low intensity of the non-homogeneous US waves that were emitted toward the tumors. A statistically significant reduction in tumor size was noticeable after the first few days of treatment leading to up to 78% reduction in tumor size in one mouse; 30–40% reduction in two other mice; and more minor reductions in the three remaining mice. Future optimization of both the US apparatus (i.e., a designated optimized apparatus for the excitation of SDT agents) and the administration regimen of **3-Ga** NPs could lead to a very promising effective pre-clinical treatment of pancreatic cancer.

## Experimental Section

EMEM growth medium, fetal bovine serum, penicillin–streptomycin, and supplements (glutamine and pyruvate) were purchased from Biological Industries (Beit-Haémek, Israel). Apo-transferrin and PBS pH 7.2 were from Sigma Aldrich. The materials used for synthesis and work-up procedures were purchased from Sigma Aldrich, Merck, Fluka, and Frutarom and used upon arrival unless otherwise stated. Deuterated solvents (Sigma Aldrich isotopes products) with a 99.5% minimum deuteration were used upon arrival. Silica gel for column chromatography (Silica Gel 60, 63–200 μm mesh) was obtained from E. Merck Ltd. Pyrrole was run through a short basic alumina column, and aldehydes were purified by vacuum distillation before use. HPLC analysis was performed on a combination of a JASCO organizer, a diode array detector MD-4010, an autosampler AS-4050, and an RHPLC pump PU-4180. The silica gel (230–400 mesh) used for column chromatography was obtained from E. Merck Ltd. A purity of >95% for **3-Ga** was determined with HPLC and UV detection at their Soret band region; a summary of HPLC results and the HPLC conditions is shown in the Supporting Information.

### Synthetic Procedures

**3-Ga** used for apo-transferrin coated NPs was prepared using a previously reported procedure.^14,33^ In short, commercially available chemicals were purchased from Sigma-Aldrich, Merck, and Chem Intel and used as received unless otherwise stated. Apo-transferrin is from Sigma-Aldrich (St. Louis, MO, US). Analytical reagent (AR)-grade solvents were used for the reactions and column chromatography. Pyrrole was subjected to filtration using a column packed with neutral aluminum oxide before use, while the remaining reagents were employed without further purification. Unless otherwise stated, synthesis was performed in ambient conditions. Produced **3-Ga** for *in vivo* studies was recrystallized in DCM/hexanes 1:1 and washed with cold hexanes for maximal purity. HPLC analysis was performed on a combination of a JASCO organizer, a diode array detector MD-4010, an autosampler AS-4050, and an RHPLC pump PU4180. Silica gel (230–400 mesh) used for column chromatography was obtained from E. Merck Ltd. Either flash or preparative thin-layer chromatography was performed to purify the compounds. A purity of >97% for **3-Ga** was determined with HPLC and UV detection at their Soret band region; a summary of the HPLC result and the HPLC condition of **3-Ga** is shown in the Supporting Information (Figure S12–S14).

### DLS and Nanosight NS300 Systems

Samples of NPs were diluted 10,000-fold in PBS. They were then analyzed using the Nanosight NS300 or the PSS Nicomp 380 DLS-ZLS analyzer in accordance with the manufacturer’s instructions.

### Cryo-TEM Analysis

Samples of NPs were prepared at a mass percentage of 1% particles in PBS, and the cryo-TEM specimens were prepared in a controlled environment vitrification system (CEVS). Cryogenic transmission electron microscopy (cryo-TEM) imaging was performed using an FEI Talos 200C, FEG-equipped cryo-dedicated high-resolution transmission electron microscope (TEM and STEM), operated at an accelerating voltage of 120 kV. Specimens were transferred into an Oxford CT-3500 Cryo-holder (Philips) or a Gatan 626DH (FEI) cryo-holder and equilibrated below −178 °C. Specimens were examined using a low-dose imaging procedure to minimize electron-beam radiation damage. Images were recorded digitally by a Gatan Multiscan 791 cooled CCD camera (Philips CM 120) or a Gatan US 1000 high-resolution CCD camera (Tecnai T12 G2), using DigitalMicrograph software.

### HPLC

HPLC analysis was performed using a MERCK HITACHI HPLC system with a diode array detector supported with HPLC Chromaster Driver for Waters Empower3 Software. Ten microliters of each sample were injected using the autosampler. Size exclusion chromatography was performed using a SephadexTM 200 10/300 GL column, with a 0.5 mL/min elution rate using sterile PBS (Sigma, sterile-filtered, isotonic, pH 7.2) as the eluent.

### Human Cancer Cell Lines

Human CRPC DU-145 cells were grown in EMEM medium (ATCC) containing 2 mM l-glutamine, supplemented with 10% fetal bovine serum (FBS, Biological Industries), 12.5 units/mL penicillin, and 6.5 μg/mL streptomycin and maintained at 37 °C under 5% CO_2_ in a humidified incubator. Cell lines were maintained up to 10 passages. Human pancreatic cancer Panc-1 cells were grown in DMEM medium (ATCC) containing the above supplementations.

### MTT

Cell death was evaluated using a colorimetric [3-(4,5-dimethyl-thiazol-2-yl)-2,5-diphenyl-tetrazolium bromide] (MTT) assay. Panc-1 cells were placed in 96-well microtiter plates at a density of 2 × 10^4^ cells/well and allowed to attach for 24 h before treatment. Cells were exposed to **3-Ga** NPs alone or with the exposure to US at 1 Hz, 0.1 w/cm^2^ for 1 min using portable Intelect Mobile Ultrasound apparatus from Chattanooga (USA) after administration of US gel (Supragel, LCH, London, UK) on the bottom of the plate for apparatus contact, covering a total of 9 wells at a time, within the radius of the transducer. Cells intended for US exposure were plated separately. Cells were incubated for 24 h post-treatment at 37 °C under 5% CO_2_. MTT was then added at a final concentration of 0.5 mg/mL for 24 h at 37 °C. After this incubation period, purple formazan salt crystals were formed by a NADP/NADPH-dependent process, in metabolically active cells. These salt crystals are insoluble in aqueous solution and were solubilized by adding a solubilizing solution (0.01 M HCl, 10% SDS) and incubating the plates for at least 6 h in a humidified atmosphere (37 °C, 5% CO_2_). The absorption of MTT was determined in a Tecan Sunrise Elisa-Reader (Switzerland) at 570/650 nm after automatic subtraction of background readings.

### Live Imaging

Panc-1 cells were plated in D × H 35 mm × 10 mm culture dishes (3 × 10^4^ cells/well) in serum-containing DMEM (phenol red-free) and allowed to attach for 24 h. **3-Ga** NPs (1 μM) were added to the medium, and cells were exposed to US 1 MHz, 0.1 w/cm^2^ for 1 min. Control cells were plated in a separate plate. Fluorescence was followed using a 40× objective (NA 1.4) and a LSM700 confocal system (Zeiss, Oberkochen, Germany) supported with Zen software. Samples were excited at 405 nm for **3-Ga** detection (MBS 405/488/555/639; (**3-Ga**) excitation = 405 nm (3%), split = LP 525 nm).

### Pharmacokinetics (PK)

Female ICR mice were housed three per cage in individual ventilated cages (IVC Cat. #1284) measuring 36.5 × 20.7 × 14.0 cm with a stainless steel top grill facilitating pelleted food and drinking water in plastic bottles; the bedding was composed of steam sterilized clean paddy husk (Envigo, Teklad, Laboratory grade, Sani-chips). Bedding material was replaced along with the cage at least twice a week. Animals were fed *ad libitum* with a commercial rodent diet (Teklad Certified Global 18% Protein Diet, Harlan cat# 2018SC). Animals had free access to sterilized drinking water. Mice are commonly used species for PK studies in accordance with the published scientific literature. The ICR strain is a well-known laboratory animal with sufficient historical data and was also used for PK determination. A total of 38 ICR female mice were used (3 for each of the six data point times, two distinct doses and two mice for the base line). Each compound was tested at two doses, and blood samples following IV administration were obtained at six time points. Three mice were sacrificed at each time point (3 × 6 = 18), and one mouse per group served for baseline values. IV injection was the preferred route of administration of the test items and the planned route of administration in humans. Blood samples were collected and processed for serum isolation by blood clotting and light centrifugation (in order to avoid precipitation of NPs). Serum samples were collected, and 100 μL of each sample (including baseline) was added to 96-well black microplates for fluorescence-based assays (Thermo Fisher, Waltham, MS, USA) for the measurements of the full emission spectra previously revealed for **3-Ga** using a BioTek (Winooski, VR, USA) plate reader, exciting the corrole at 420 nm. Emission intensities were incorporated into a standard curve function (for serum concentration derivation) generated using emission intensities of *in situ* added **3-Ga** NPs to baseline mouse serum samples measured under the same conditions.

### Xenograft Model Based on Human Prostate Cancer DU-145 Cells

Nude mice (FOX1^nu^, Envigo, Indianapolis, IN, USA) handling was performed according to the guidelines of the National Institutes of Health (NIH) and the Association for Assessment and Accreditation of Laboratory Animal Care (AAALAC). The study was conducted in one cycle. On the first day of the study, mice were injected into their right flank with 10^6^ DU-145 cells in 100 μL of PBS:Matrigel (1:1, Life Sciences, Chicago. IL, USA). Tumor volume was first determined upon tumor appearance and twice a week thereafter until study termination. Once tumors reached a volume of 85–115 mm^3^, mice were distributed into six groups with similar mean tumor volume, consisting of seven or nine mice per group (in accordance with the displayed experiment: *n* = 7 or *n* = 9). IV treatments were initiated with **3-Ga** NPs for 2 weeks in accordance with the timeline depicted in Scheme 1. Following treatment, mice were monitored for body weight and tumor size for two additional weeks. Before termination, animals were weighed, tumor volumes were determined, and mice were sacrificed by CO_2_ asphyxiation followed by tumor excision, weighing, and fixation in formalin.

### Xenograft Mouse Model Based on Human Pancreatic Cancer Panc-1 Cells and IVIS Imaging

Nude mice (FOX1^nu^, Envigo, Indianapolis, IN, USA) handling was performed according to guidelines of the NIH and AAALAC. Mice were housed and treated as detailed above. Animals intended for US exposure received US waves of 1 MHz, 1 w/cm^2^ 50% duty cycle for 2 min using portable Intelect Mobile Ultrasound apparatus from Chattanooga (USA) after topical administration of US gel (Supragel, LCH, London, UK) for apparatus contact and in accordance with the timeline schedule depicted in Scheme 2. Following treatment, mice were monitored for two additional weeks for body weight and tumor size. Before termination, animals were weighed, tumor volumes were determined, and mice were sacrificed using CO_2_ asphyxiation followed by tumor excision, weighing, and fixation in formalin. To obtain real-time imaging of **3-Ga** during systemic delivery, mice received a single i.v. injection of 5 mg/kg **3-Ga** NPs and were imaged 2.5 h after administration using a real-time *in vivo* fluorescence image acquisition using an IVIS Lumina X5 (Perkin Elmer, Waltham, MA, US). A 420 nm laser light was used for the excitation of corrole conjugates, delivered onto the mice through mirrors, enabling uniform excitation of the specimen. The emitted light from the mice was imaged passing through standard interference filters (Chroma, 790 nm 40 nm) before arriving onto a cooled CCD camera (−90 °C) located on top of the light-tight imaging chamber.

### Statistical Analysis

Data were expressed as mean values ± S.E.M and compared between experimental groups with the use of one-way analysis of variance followed by Tukey’s post-hoc test unless otherwise specified (Analyze-it software for Windows Excel, Leeds, UK). Probability values of *p* < 0.05 were considered statistically significant.

## Supporting Information Available

Additional synthetic scheme; experimental details and compound characterization; DLS, cryo-TEM, ^1^H and ^19^F NMR spectra, and HPLC trace of **3-Ga**; biological data comprising **3-Ga** NP PK analysis, xenograft model based on human prostate cancer DU-145 cells, individual tumor histological evaluation, xenograft mouse model based on human pancreatic cancer Panc-1 cells, and IVIS imaging (PDF)

## Abbreviations

AUC: area under the curve
AAALAC: Laboratory Animal Care
ADT: androgen deprivation therapy
CRPC: castration-resistant prostate cancer
DMSO: dimethyl sulfoxide
HER: human epidermal growth factor receptor
HIFU: high intensity focused ultrasound
hERG: human ether-a-go-go related gene
mCRPC: metastatic castration-resistant prostate cancer
MTD: maximal tolerated dose
NPs: nanoparticles
Tf: apo-transferrin
PBS: phosphate buffered saline
ROI: region of interest
ROA: route of administration
ROS: reactive oxygen species
SDT: sonodynamic therapy
TGI: tumor growth inhibition
TfR: transferrin receptor
US: ultrasound
