# Supplementary material for: Corrole Nanoparticles for Chemotherapy of Castration-Resistant Prostate Cancer and as Sonodynamic Agents for Pancreatic Cancer Treatment
Source: J Med Chem. 2022 Dec 14;66(1):766–76. doi: 10.1021/acs.jmedchem.2c01662 (PMC9841519; doi:10.1021/acs.jmedchem.2c01662)
Supplement: Supplementary file 1 — jm2c01662_si_001.pdf [file jm2c01662_si_001.pdf]

# Supporting Information

# Corrole nanoparticles for chemotherapy of castration resistant prostate cancer and as sonodynamic agents for pancreatic cancer treatment

Matan Soll [a], Vinay K. Sharma [a], Sally Khoury [a], Yehuda G. Assaraf [b*] and Zeev Gross [a*]

[a]Schulich Faculty of Chemistry, Technion – Israel Institute of Technology, Haifa 3200003, Israel

[b]The Fred Wyszkowski Cancer Research Laboratory, [2] Department of Biology, Technion-Israel Institute of Technology, Haifa 3200003, Israel

**Corresponding Authors**

* Zeev Gross: chr10zg@technion.ac.il
* Yehuda G. Assaraf: assaraf@technion.ac.il

**Table of Contents**


| | | |
|---|---|---|
| Figure S1 | **3-Ga NPs PK analysis** | S3 |
| Figure S2 | **Xenograft model of nude mice ($Foxn1^{nu}$) implanted with DU-145 hormone refractive castration resistance cell line tumors.** | S4 |
| Figure S3 | **Body Weight mesurments of Xenpgraft prostate cancer model experiments** | S5 |
| Figure S4 | **Reprasentative images of tumor bearing nude mice at end of the experiment before euthanization** | S6 |
| Table S1 | **Individual tumor histological evaluation** | S7 |
| Figure S5 | **Representative H&E pictures of the tumors margins show some inflammatory and necrotic changes per group (X10)** | S9 |
| Figure S6 | **Pilot of xenograft model of nude mice ($Foxn1^{nu}$) implanted with Panc-1 pancreatic cancer cell line tumors.** | S10 |
| Figure S7 | **Xenograft model of nude mice (Foxn1 nu) implanted with Panc-1 pancreatic cancer cell line tumors of mice** | S10 |
| Figure S8 | **Specificity of 3-Ga NPs** | S11 |
| Figure S9 | **AMES MPF** | S12 |
| Figure S10 | **The effects of test items on hERG tail currents** | S13 |
| Figure S11 | **Structure and formulation assessments of 3-Ga NPs** | S14 |
| Figure S12 | **$^{1}H$ NMR spectrum of 3-Ga in $CDCl_3$. Asterisk represents the solvent peak.** | S15 |
| Figure S13 | **$^{19}F$ NMR spectrum of 3-Ga in $CDCl_3$.** | S16 |
| Figure S14 | **HPLC chromatogram (column: C18 silica gel, flow rate: 1 mL/min, eluent: MeOH:water = 88:12, pH 7.4) of 3-Ga** | S17 |
| **Methods** | **Tf coated NPs formulation** | S17 |

(A)

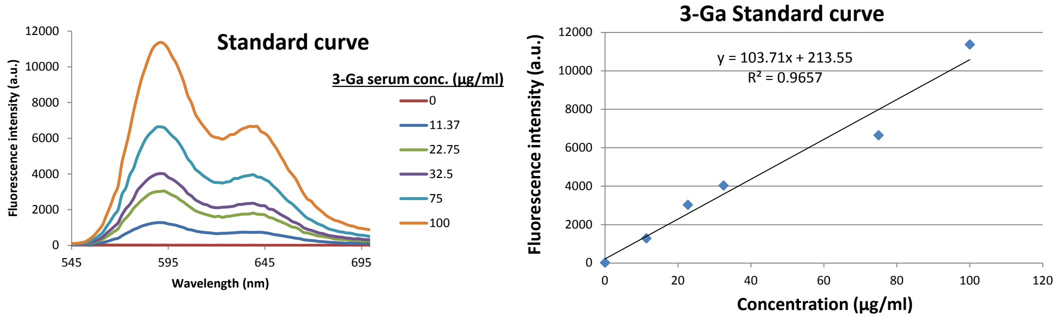


(B)

| Time (min) | Fluorescence intensity (a.u.) | | | Derived concentration (µg/ml) | | | Concentration (µg/ml) | STDev | SEM | ln(Cp) | Be^-bt | Ae^-at |
|---|---|---|---|---|---|---|---|---|---|---|---|---|
| 5 | 52045 | 39840 | 64659 | 4042.019 | 3108.486 | 5006.834 | 4052.446331 | 775.0323 | 447.4651 | 8.307076 | 803.2064 | 3249.24 |
| 30 | 41755 | 40235 | 49582 | 3254.96 | 3138.699 | 3853.629 | 3415.762837 | 313.2352 | 180.8465 | 8.136156 | 799.2004 | 2616.562 |
| 60 | 44204 | 50809 | 41714 | 3442.279 | 3947.48 | 3251.824 | 3547.194177 | 293.5298 | 169.4695 | 8.173912 | 794.4195 | 2752.775 |
| 240 | 91910 | 103560 | 94751 | 709.12 | 798.2282 | 730.8502 | 746.0661109 | 37.936 | 21.90236 | 6.614814 | 766.3291 | -20.263 |
| 1440 | 67993 | 74782 | 92564 | 526.1844 | 578.1119 | 714.1223 | 606.139539 | 79.24361 | 45.75132 | 6.40711 | 602.8158 | 3.323721 |
| 2880 | 49979 | 52758 | 57766 | 388.3995 | 409.6554 | 447.9605 | 415.338458 | 24.64548 | 14.22908 | 6.029094 | 451.9681 | -36.6297 |

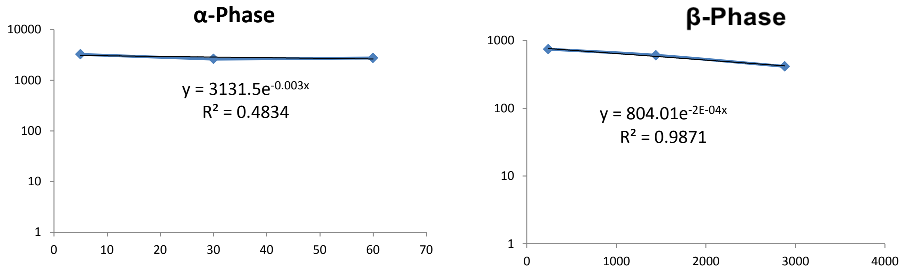


(C)

| Time (min) | Fluorescence intensity (a.u.) | | | Derived concentration (µg/ml) | | | Concentration (µg/ml) | STDev | SEM | ln(Cp) | Be^-bt | Ae^-at |
|---|---|---|---|---|---|---|---|---|---|---|---|---|
| 5 | 54613 | 62180 | 50227 | 423.8439 | 481.7221 | 390.2964 | 431.9541329 | 37.7624 | 21.80213 | 6.068319 | 198.7516488 | 233.2025 |
| 30 | 42121 | 42523 | 41224 | 328.2955 | 331.3703 | 321.4345 | 327.0334251 | 4.15326 | 2.397886 | 5.790062 | 197.2665874 | 129.7668 |
| 60 | 39852 | 36656 | 34348 | 310.9404 | 286.495 | 268.8416 | 288.7589873 | 17.26117 | 9.965742 | 5.665592 | 195.4991535 | 93.25983 |
| 240 | 23312 | 21987 | 25236 | 184.4298 | 174.2952 | 199.146 | 185.9569884 | 10.20263 | 5.89049 | 5.225515 | 185.2221748 | 0.734814 |
| 1440 | 20698 | 13712 | 18321 | 164.4359 | 111.0016 | 146.2548 | 140.5640967 | 22.18248 | 12.80706 | 4.945664 | 129.2251264 | 11.33897 |
| 2880 | 12056 | 12413 | 10739 | 98.33525 | 101.0659 | 88.26182 | 95.88763959 | 5.506295 | 3.179061 | 4.563177 | 83.89416378 | 11.99348 |

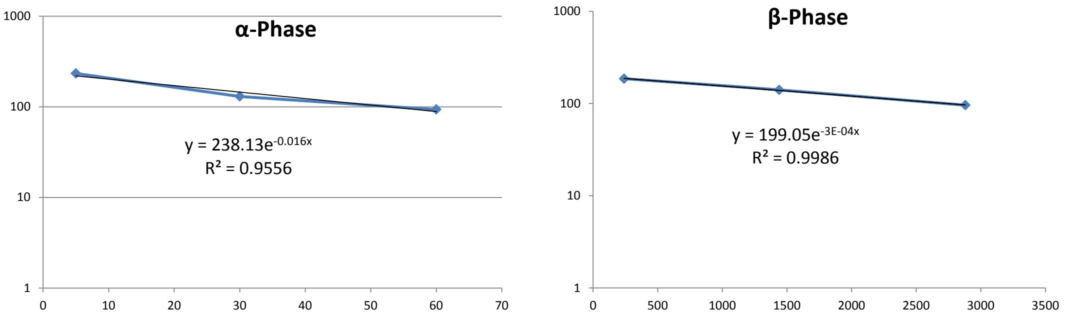


**Figure S1 3-Ga NPs PK analysis:(A)** standard curve estimation of **3-Ga** NPs concentration in mice serum vs. Flurescnece readout. **(B)** Raw data of **3-Ga** NPs serum concentration at different time points as extracted from sirculation after IV administration of 10 mg/Kg NPs. Estimates of alpha and beta phase values derivation are depected as derived from exponential curve; and **(C)** Raw data of **3-Ga** NPs serum concentration at different time points as extracted from sirculation after IV administration of 2 mg/Kg NPs. Estimates of alpha and beta phase values derivation are depected as derived from exponential curve.

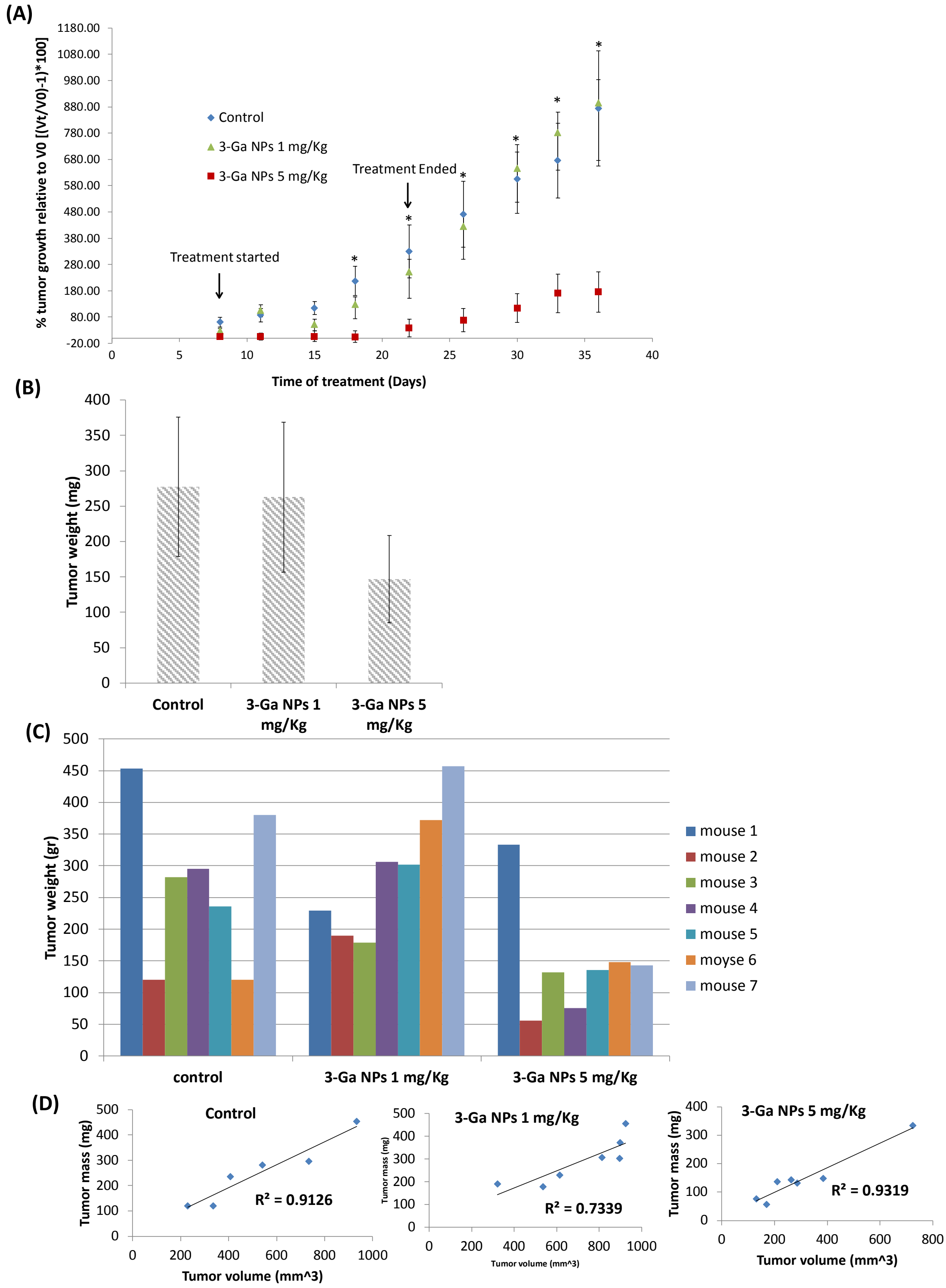


**Figure S2 (A) Xenograft model of nude mice (Foxn1$^{nu}$) implanted with DU-145 hormone refractive castration resistance cell line tumors.** Tumors were measured for width (W) and length (L) using caliper and volume (V) was calculated according to the following equation: $V=LW^2/2$, data was normalized (relative to initial tumor size at day 5) and plotted. Results represent mean ±SEM of all mice in each group (n=7, *$p<0.05$ at 3-Ga 10 mg/Kg vs. control, according to t-Test). **(B)** Average tumor weight at end point. **(C)** Individual tumor weight distribution at end point. **(D)** tumor volume to tumor weight at end

point relative correlation coeeificient estimation (i.e., substantiation of tumor volume measurments throughout the experiment).

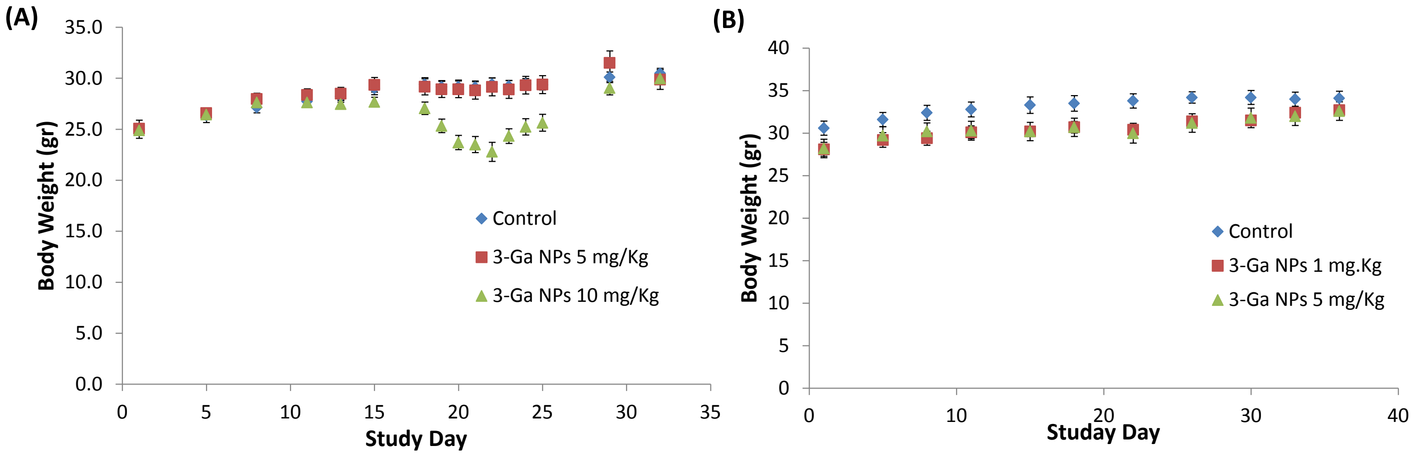


**Figure S3 Body Weight mesurments of Xenpgraft prostate cancer model experiments: (A)** body weight measurments of DU-145 cell line xenograft model depicted in Figure 2 with N=9. Results represent mean ±SEM of all mice in each group; **(B)** body weight measurments of DU-145 cell line xenograft model depicted in Figure 3 (main text) with N=9. Results represent mean ±SEM of all mice in each group.

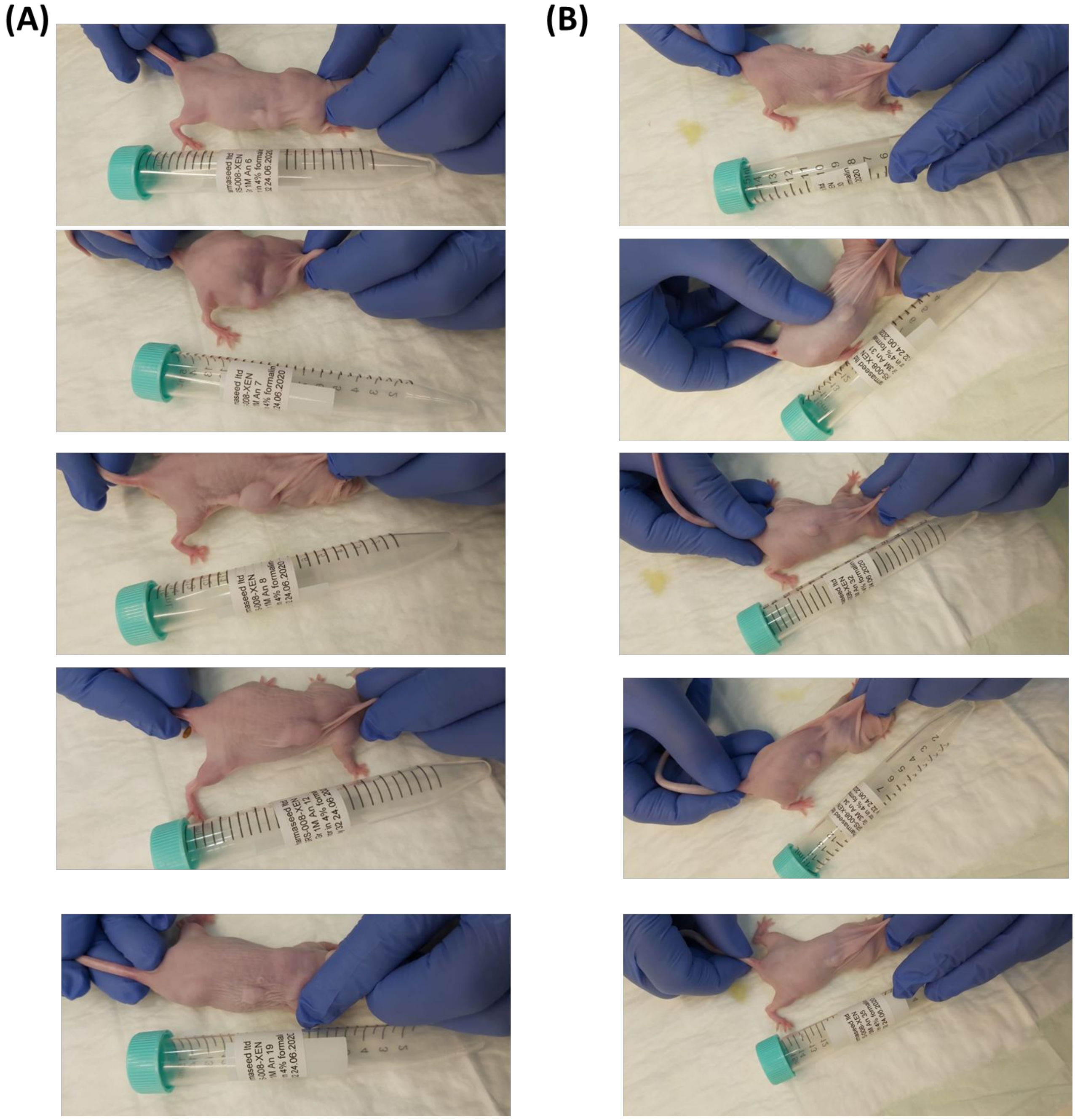


**Figure S4 Reprasentative images of tumor bearing nude mice at end of the experiment before euthanization: (A)** control and **(B) 3-Ga** NPs 10 mg/Kg treated mice depicted in Figure 3 (main text) with N=9.

## Table S1 Individual tumor histological evaluation

| Animal No. | Animal Group | Inflammation score | % lymphocytes | % macrophages | % neutrophils | Mitotic index | Necrosis score | Tumor matrix score | Nuclear pleomorphism score | MNGC | Vascular invasion |
|---|---|---|---|---|---|---|---|---|---|---|---|
| 2 | Control | 1 | 60 | 30 | 10 | 2 | 2 | 1 | 3 | Y | N |
| 3 | Control | 1 | 60 | 30 | 10 | 2 | 1 | 1 | 3 | Y | N |
| 5 | Control | 1 | 60 | 20 | 20 | 2 | 2 | 1 | 3 | Y | Y |
| 6 | Control | 1 | 60 | 30 | 10 | 2 | 2 | 1 | 3 | Y | N |
| 7 | Control | 1 | 40 | 50 | 10 | 2 | 2 | 1 | 3 | Y | N |
| 10 | Control | 1 | 40 | 50 | 10 | 2 | 2 | 1 | 3 | Y | N |
| 18 | Control | 1 | 50 | 40 | 10 | 2 | 2 | 1 | 3 | Y | N |
| | **Mean:** | 1.00 | | | | 2.00 | 1.86 | 1.00 | 3.00 | | |
| | **Std:** | 0.49 | | | | 0.00 | 0.58 | 0.00 | 0.00 | | |
| 47 | **3-Ga** 5 mg/Kg | 2 | 40 | 40 | 20 | 2 | 1 | 1 | 3 | Y | N |
| 48 | **3-Ga** 5 mg/Kg | 1 | 50 | 40 | 10 | 2 | 1 | 1 | 3 | Y | N |
| 49 | **3-Ga** 5 mg/Kg | 2 | 50 | 40 | 10 | 2 | 2 | 1 | 3 | Y | N |
| 50 | **3-Ga** 5 mg/Kg | 2 | 40 | 40 | 20 | 2 | 2 | 1 | 3 | Y | N |
| 52 | **3-Ga** 5 mg/Kg | 3 | 40 | 40 | 20 | 2 | 3 | 1 | 3 | Y | N |
| 54 | **3-Ga** 5 mg/Kg | 2 | 50 | 40 | 10 | 2 | 2 | 1 | 3 | Y | N |
| 55 | **3-Ga** 5 mg/Kg | 3 | 70 | 20 | 10 | 2 | 3 | 1 | 3 | Y | N |
| | **Mean:** | 2.14 | | | | 2.00 | 2.00 | 1.00 | 3.00 | | |
| | **Std:** | 0.69 | | | | 0.00 | 0.82 | 0.00 | 0.00 | | |
| 56 | **3-Ga** 10 mg/Kg | 2 | 50 | 40 | 10 | 2 | 1 | 1 | 3 | Y | N |
| 57 | **3-Ga** 10 mg/Kg | 3 | 60 | 30 | 10 | 2 | 2 | 1 | 3 | Y | N |
| 59 | **3-Ga** 10 mg/Kg | 3 | 50 | 40 | 10 | 2 | 2 | 1 | 3 | Y | N |
| 62 | **3-Ga** 10 mg/Kg | 3 | 60 | 30 | 10 | 2 | 2 | 1 | 3 | Y | N |

| Animal No. | Animal Group | Inflammation score | % lymphocytes | % macrophages | % neutrophils | Mitotic index | Necrosis score | Tumor matrix score | Nuclear pleomorphism score | MNGC | Vascular invasion |
|---|---|---|---|---|---|---|---|---|---|---|---|
| 63 | **3-Ga** 10 mg/Kg | 2 | 50 | 40 | 10 | 2 | 1 | 1 | 3 | Y | N |
| | **Mean:** | 2.6 | | | | 2.00 | 1.71 | 1.00 | 3.00 | | |
| | **Std:** | 0.49 | | | | 0.00 | 0.49 | 0.00 | 0.00 | | |

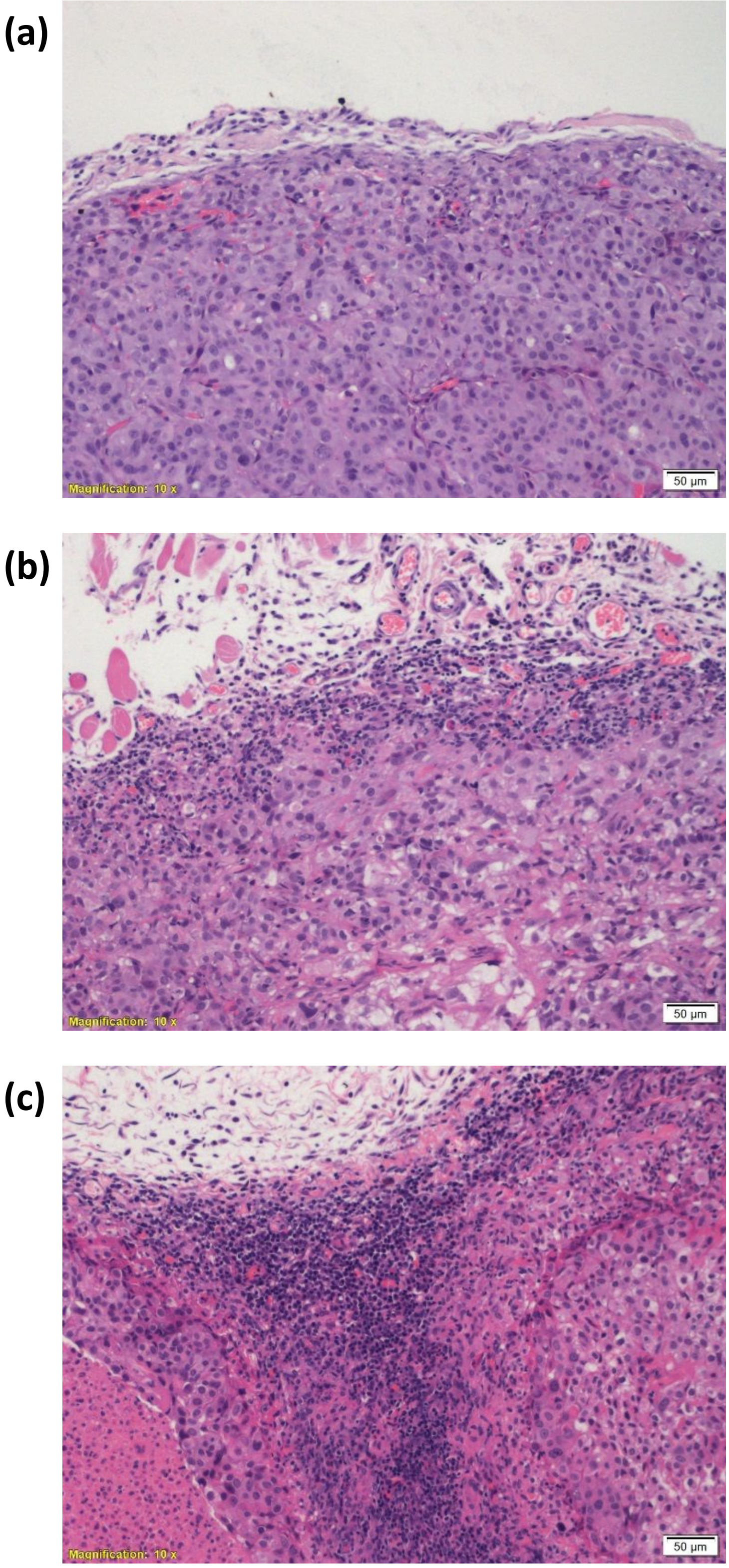

**Figure S5 Representative H&E pictures of the tumors margins show some inflammatory and necrotic changes per group (X10):** (a) Control, (b) **3-Ga** NPs 5 mg/kg and (c) **3-Ga** Nps 10 mg/Kg

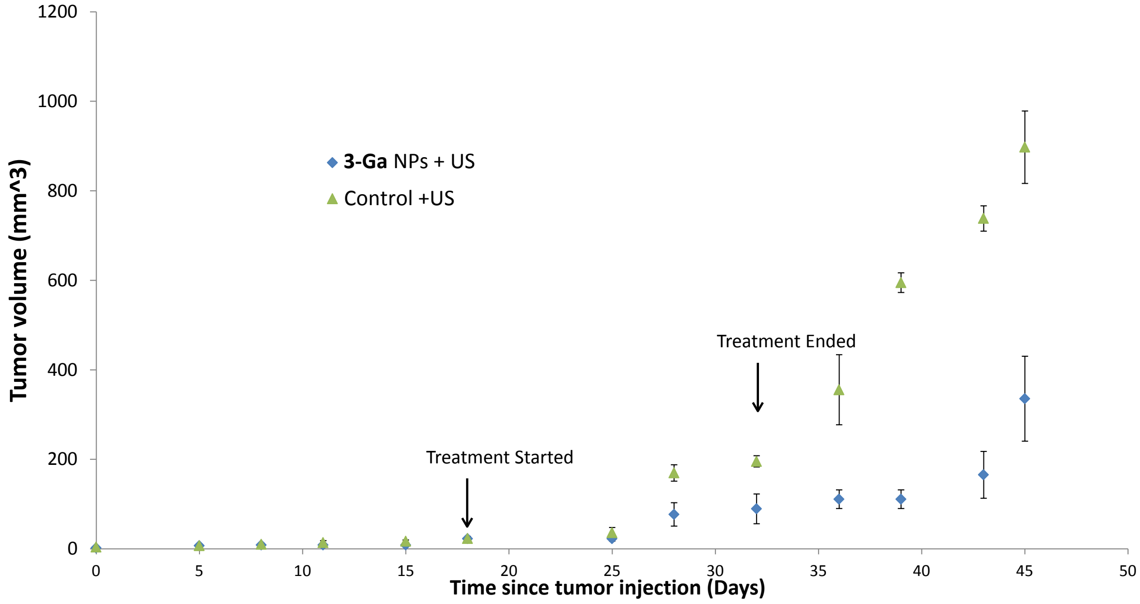


**Figure S6 Pilot of xenograft model of nude mice ($Foxn1^{nu}$) implanted with Panc-1 pancreatic cancer cell line tumors.** Tumors were measured for width (W) and length (L) using caliper and volume (V) was calculated according to the following equation: $V=LW^2/2$. Results represent mean ±SEM of all mice in each group (n=3).

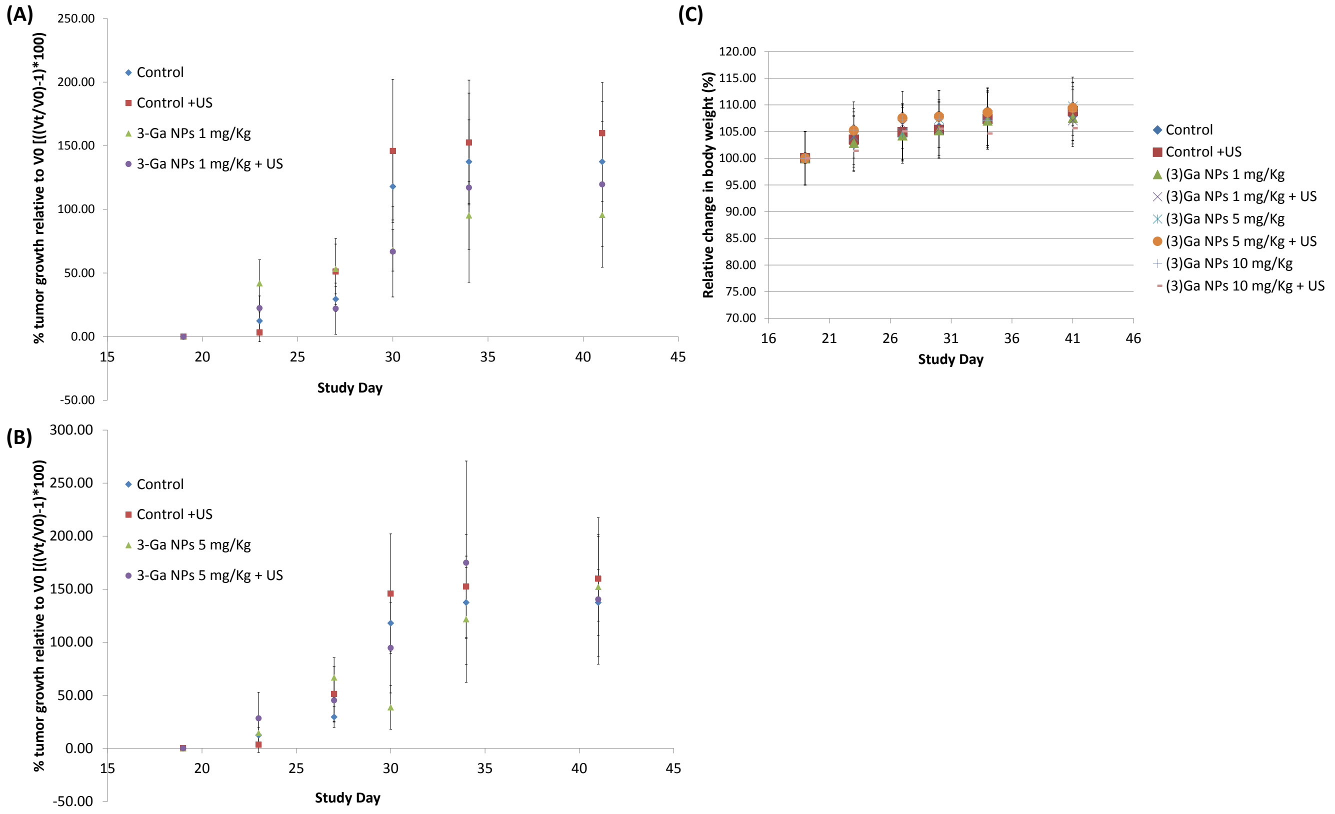


**Figure S7 Xenograft model of nude mice (Foxn1 nu) implanted with Panc-1 pancreatic cancer cell line tumors of mice:** treated with (A) 1 mg/Kg of (3)Ga NPs with and without ultrasound exposure relative to Control (with and without ultrasound); and (B) 5 mg/Kg of (3)Ga NPs with and without ultrasound exposure relative to Control (with and without ultrasound). Tumors were measured for width (W) and length (L) using caliper and volume (V) was calculated according to the following equation:

V=LW2/2, data was normalized (relative to initial tumor size at day 5) and plotted. Results represent mean ±SEM of all mice in each group (n=7). (C) Changes in body weight (gr) during the study. Data was plotted as percentage of change, relative to the initial weight. Results represent mean ±SEM of all mice in each group.

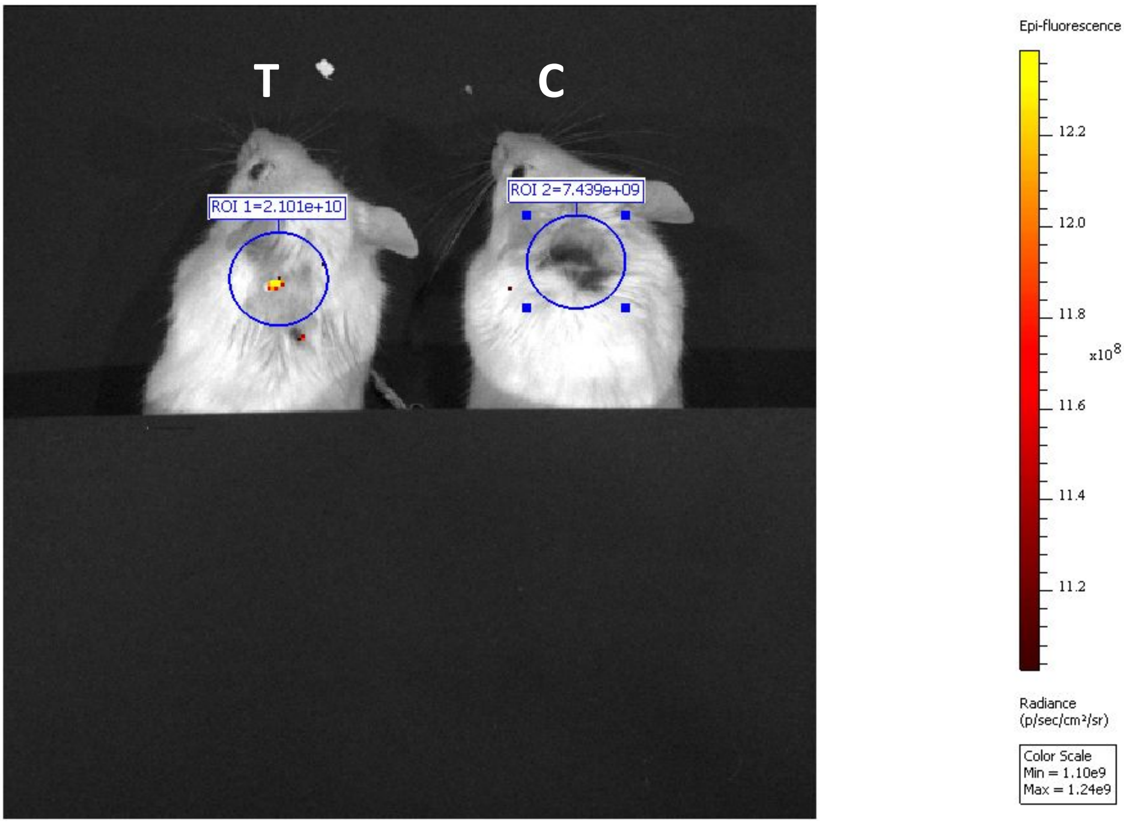


**Figure S8 Specificity of 3-Ga NPs:** NSG mice bearing human Panc-1 tumors ( 65 cubic mm) received a single IV injection of either **3-Ga** NPs (**T,** ROI 1, 5 mg/Kg) or Vehicle (**C**, ROI 1, roughly 120 µL) and were imaged at 2.5 hr post injection using a noninvasive small animal fluorescence imaging system. ROI desclose estimation of fluorescnece intensity at each treated mice within the enclosed ROI.

3-Ga NPS

TA98 -S9 Assay Date: 12/30/2019

| Conc. (µg/ml) | n | Mean # positive wells | SD | Base-line | Fold increase over baseline | Binomial B-value |
|---|---|---|---|---|---|---|
| 0 | 12 | 2.33 | 0.89 | 3.22 | | |
| 15.625 | 3 | 0.00 | 0.00 | | 0.00 | 0.0008 |
| 31.25 | 3 | 0.00 | 0.00 | | 0.00 | 0.0008 |
| 62.5 | 3 | 0.00 | 0.00 | | 0.00 | 0.0008 |
| 125 | 3 | 0.00 | 0.00 | | 0.00 | 0.0008 |
| 250 | 3 | 0.00 | 0.00 | | 0.00 | 0.0008 |
| 500 | 3 | 0.00 | 0.00 | | 0.00 | 0.0008 |
| 2-NF | 12 | 48.00 | 0.00 | | **14.90** | **1.0000** |
| *Limit for "Pass":* | | | | | *3.00* | *0.99* |

3-Ga NPS

TA98 +S9 Assay Date: 12/30/2019

| Conc. (µg/ml) | n | Mean # positive wells | SD | Base-line | Fold increase over baseline | Binomial B-value |
|---|---|---|---|---|---|---|
| 0 | 12 | 2.67 | 1.23 | 3.90 | | |
| 15.625 | 3 | 2.67 | 0.58 | | 0.68 | 0.5926 |
| 31.25 | 3 | 3.00 | 0.00 | | 0.77 | 0.7203 |
| 62.5 | 3 | 2.33 | 0.58 | | 0.60 | 0.4490 |
| 125 | 3 | 1.33 | 1.15 | | 0.34 | 0.0932 |
| 250 | 3 | 0.33 | 0.58 | | 0.09 | 0.0025 |
| 500 | 3 | 0.00 | 0.00 | | 0.00 | 0.0003 |
| 2-AA | 12 | 47.42 | 1.08 | | **12.17** | **1.0000** |
| *Limit for "Pass":* | | | | | *3.00* | *0.99* |

3-Ga NPS

TA100 -S9 Assay Date: 12/30/2019

| Conc. (µg/ml) | n | Mean # positive wells | SD | Base-line | Fold increase over baseline | Binomial B-value |
|---|---|---|---|---|---|---|
| 0 | 12 | 3.83 | 2.37 | 6.20 | | |
| 15.625 | 3 | 0.00 | 0.00 | | 0.00 | 0.0000 |
| 31.25 | 3 | 0.00 | 0.00 | | 0.00 | 0.0000 |
| 62.5 | 3 | 0.00 | 0.00 | | 0.00 | 0.0000 |
| 125 | 3 | 0.00 | 0.00 | | 0.00 | 0.0000 |
| 250 | 3 | 0.00 | 0.00 | | 0.00 | 0.0000 |
| 500 | 3 | 0.00 | 0.00 | | 0.00 | 0.0000 |
| 4-NQO | 12 | 46.92 | 1.31 | | **7.57** | **1.0000** |
| *Limit for "Pass":* | | | | | *2.00* | *0.99* |

3-Ga NPS

TA100 +S9 Assay Date: 12/30/2019

| Conc. (µg/ml) | n | Mean # positive wells | SD | Base-line | Fold increase over baseline | Binomial B-value |
|---|---|---|---|---|---|---|
| 0 | 12 | 6.33 | 1.30 | 7.64 | | |
| 15.625 | 3 | 10.33 | 2.52 | | 1.35 | **0.9980** |
| 31.25 | 3 | 12.00 | 2.00 | | 1.57 | **1.0000** |
| 62.5 | 3 | 10.67 | 2.52 | | 1.40 | **0.9990** |
| 125 | 3 | 10.33 | 2.08 | | 1.35 | **0.9980** |
| 250 | 3 | 7.67 | 4.04 | | 1.00 | 0.8650 |
| 500 | 3 | 5.00 | 1.00 | | 0.65 | 0.1964 |
| 2-AA | 12 | 48.00 | 0.00 | | **6.29** | **1.0000** |
| *Limit for "Pass":* | | | | | *2.00* | *0.99* |

**Figure S9 AMES MPF:** indevidual data sets.

(A)

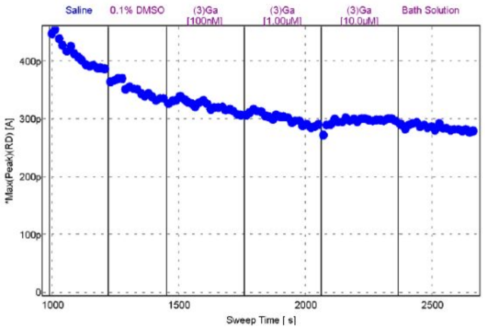


(C)

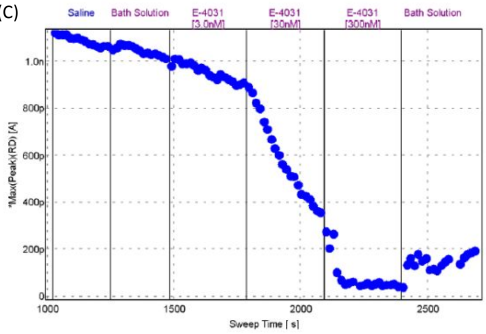


(B)

| Conc. (μM) | % Inhibition | | | Mean | Sem | N |
|---|---|---|---|---|---|---|
| 0.1 | 7.56 | 4.59 | 7.21 | **6.45** | **0.94** | **3** |
| 1 | 10.11 | 0.42 | 13.97 | **8.17** | **4.03** | **3** |
| 10 | 14.81 | -4.88 | 11.02 | **6.98** | **6.03** | **3** |

(D)

| Conc. (nM) | % Inhibition | | | | Mean | Sem | N |
|---|---|---|---|---|---|---|---|
| **3** | 17.54 | 9.39 | 11.27 | 13.26 | **12.87** | **1.75** | **4** |
| **30** | 57.81 | 32.12 | 62.82 | 65.84 | **54.65** | **7.69** | **4** |
| **300** | 98.73 | 96.34 | 95.34 | 95.60 | **96.50** | **0.77** | **4** |

(E)

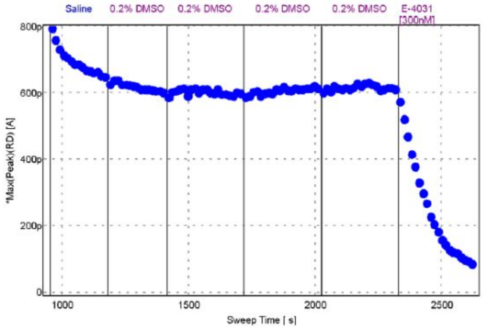


(F)

| | % Inhibition | | | Mean | Sem | N |
|---|---|---|---|---|---|---|
| **0.2% DMSO** | 1.53 | 6.10 | 5.73 | **4.45** | **1.47** | **3** |
| **0.2% DMSO** | -1.72 | 6.10 | 4.90 | **3.10** | **2.43** | **3** |
| **0.2% DMSO** | -1.37 | 5.28 | 6.17 | **3.36** | **2.38** | **3** |
| **0.3 μM E-4031** | 84.36 | 89.05 | 96.01 | **89.81** | **3.38** | **3** |

**Figure S10 The effects of test items on hERG tail currents: (A)** Example of the Effect of **3-Ga** on hERG tail current; **(B)** Individual Data for **3-Ga; (C)** Example of the Effect of **E-4031** on hERG Tail Currents; **(D)** Individual Data of **E-4031; (E)** Example of the Effect of **E-4031** on hERG Tail Currents; **(F)** Individual Data of **E-4031and DMSO.**

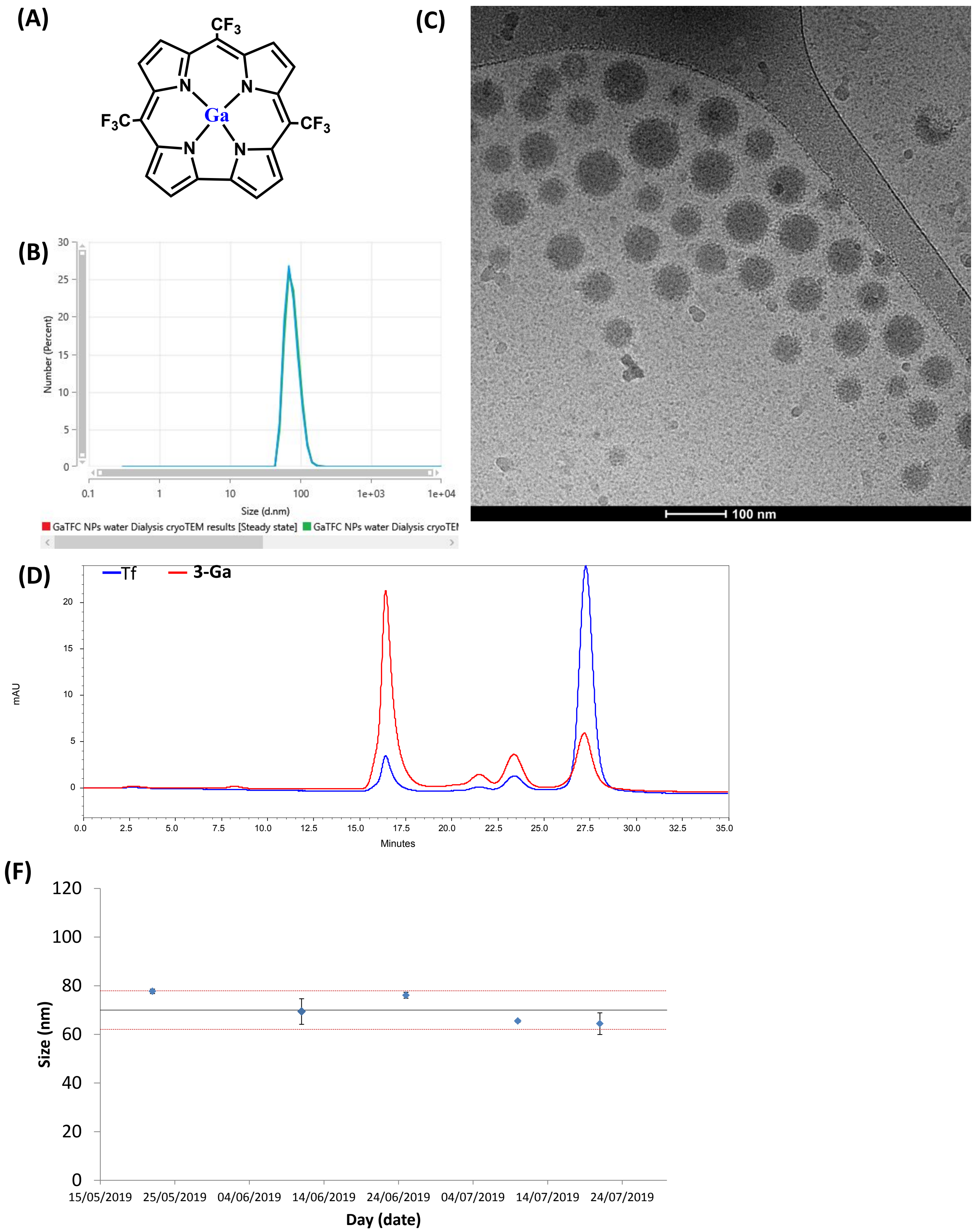


**Figure S11 Structure and formulation assessments of 3-Ga NPs: (A)** Molecular structure of 5,10,15-tris(trifluoromethyl)corrole and its gallium(III) complex (**3-Ga**); **(B)** DLS analysis of pure newly formulated **3-Ga**/Tf NPs with **77.7**±1.905 nm size and 0.0593 polydispersity; **(C)** Representative Cryo-TEM imaging of 3-Ga/Tf NPs newly formulated for *in vivo* experiment after formulation optimisation

of homogenicity and purity; **(D)** HPLC chromatogram (Sephadex TM 200 10/300 GL column) for the detection of apo-transferrin (Tf) with **3-Ga** in Tf/corrole NPs. Tf was detected at 280 nm (blue line) and corrole was detected at 422 nm (red line); **(F)** Repetative foemulation of **3-Ga**/Tf NPs under optimised conditions for use in *in vivo* experiments as depected by mean size of NPs at each data point as detetcted by DLS with standard diviation bars. Black line depetcs wanted target of 70 nm with red lines depecting lower and uper bound limits in accordance with manufacturing standards (6σ).

**$^1$H NMR (400 MHz, $CDCl_3$):**

δ (ppm) = 9.65 (td, *J* = 5.0, 2.5 Hz, 2H), 9.57 (td, *J* = 5.5, 2.8 Hz, 2H), 9.45 (td, *J* = 5.6, 2.8 Hz, 2H), 9.24 (d, *J* = 4.3 Hz, 2H), 7.05 (tt, *J* = 7.7, 1.6 Hz, 2H), 6.49 – 6.37 (m, 4H), 5.42 (d, *J* = 4.6 Hz, 4H).

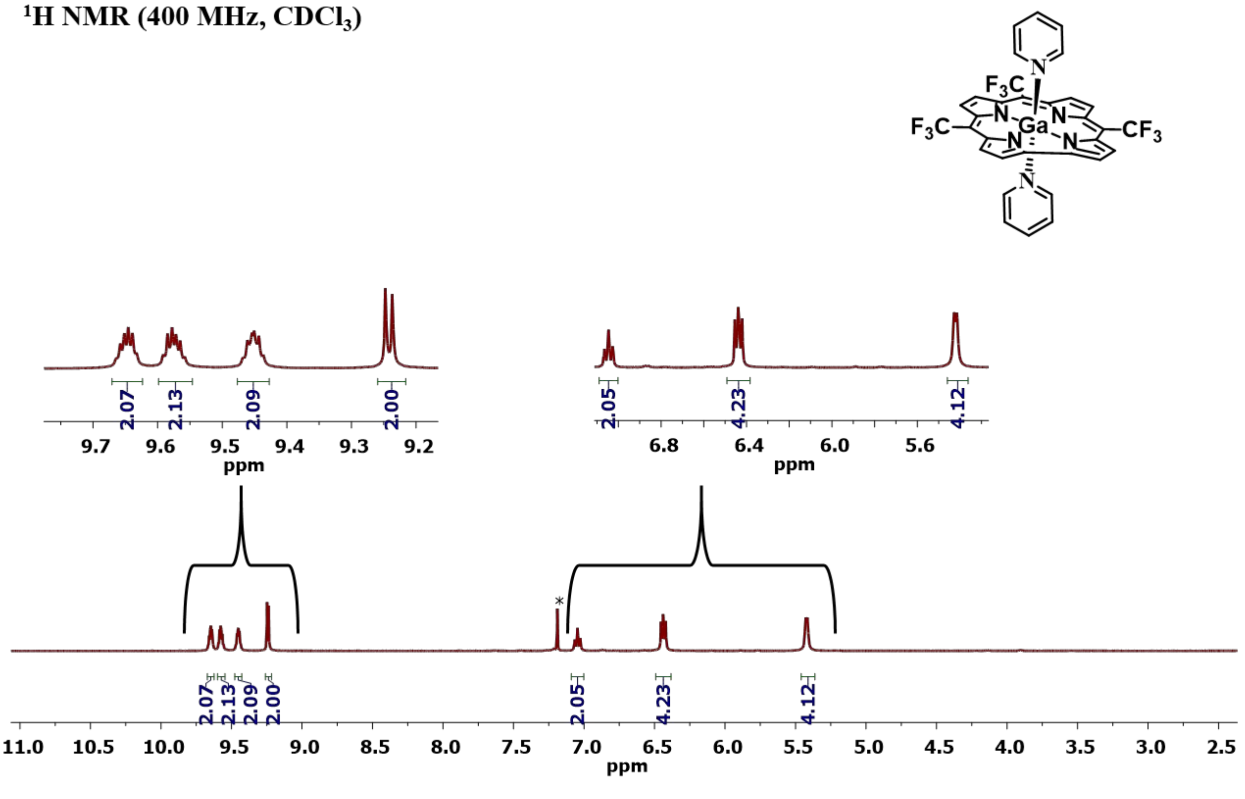


**Figure S12 $^1$H NMR spectrum of 3-Ga in $CDCl_3$. Asterisk represents the solvent peak.**

## $^{19}F$ NMR (400 MHz, $CDCl_3$)

δ (ppm) = -38.15 (s), -41.14 (s).

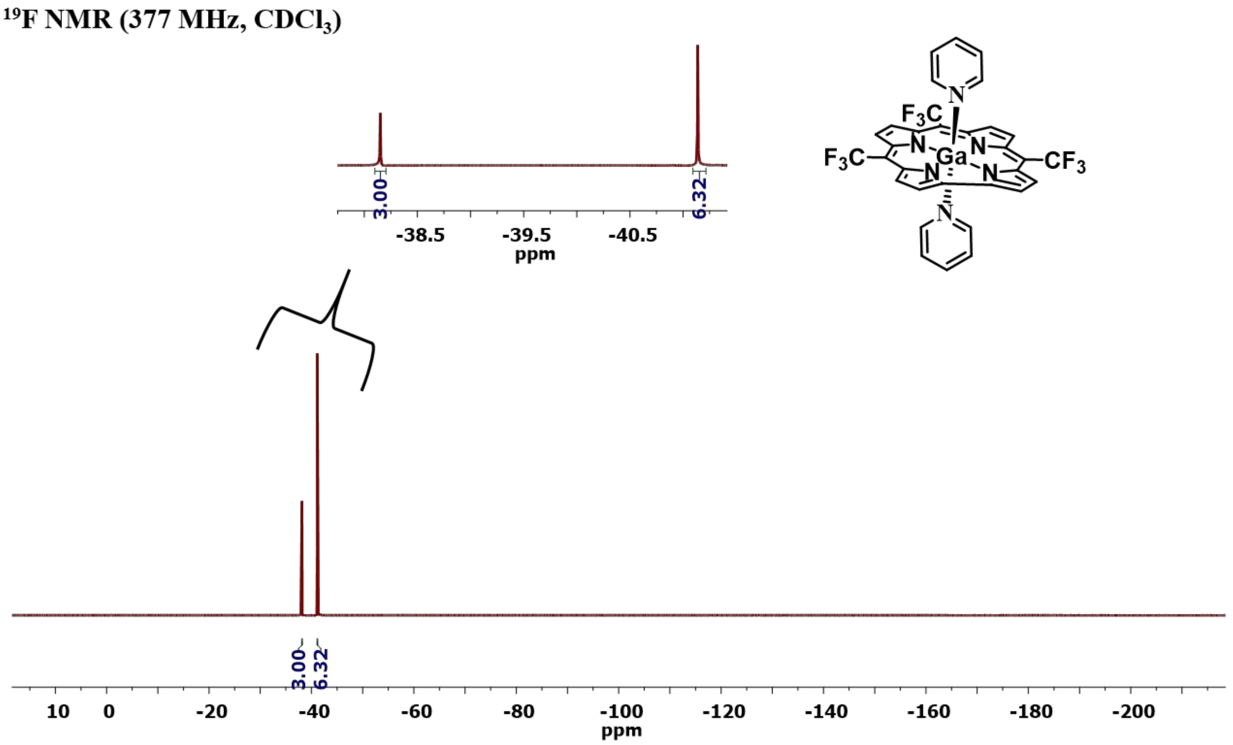


**Figure S13 $^{19}F$ NMR spectrum of 3-Ga in $CDCl_3$.**

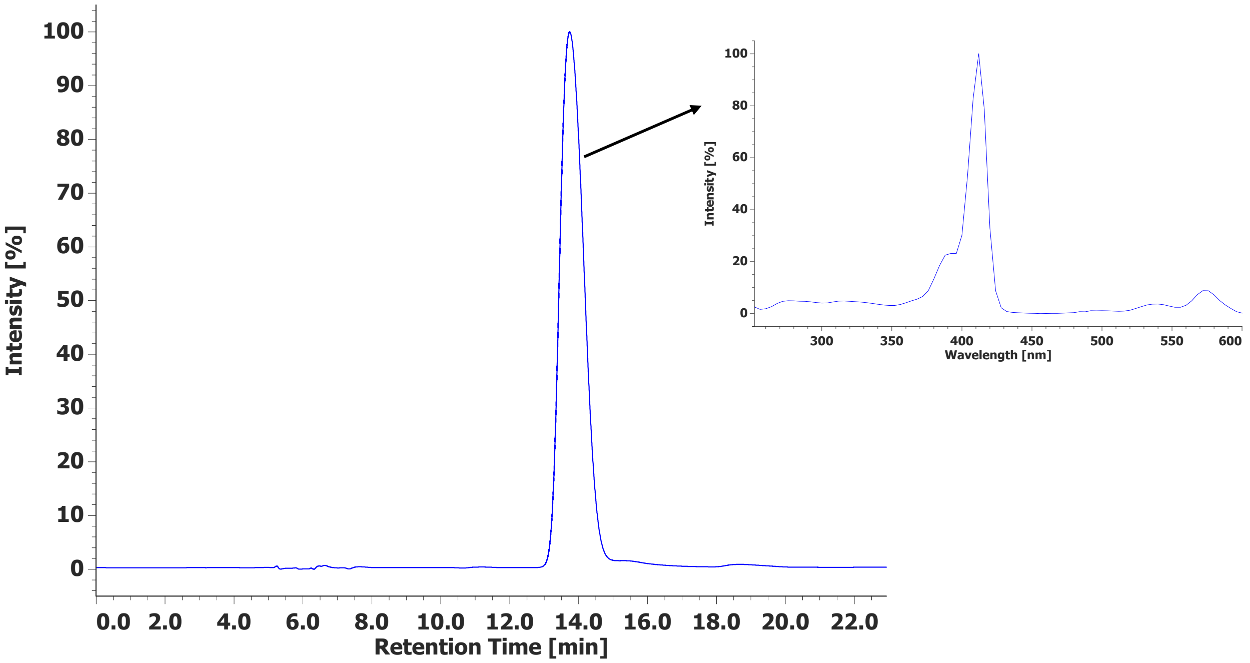


**Figure S14 HPLC chromatogram (column: C18 silica gel, flow rate: 1 mL/min, eluent: MeOH:water = 88:12, pH 7.4) of 3-Ga.** Inset shows the UV/vis spectrum corresponding to the peak.

**Tf coated NPs formulation**

A solution of 100 µL of 1 mM corrole dissolved in DMSO was added under vigorous stirring to 800 µL of deionized water (in a glass vial) using a 50 µL Hamilton syringe with the needle tip placed close to the micro stirring bar. Standard magnetic stirrer plates were used. No sign of precipitation was observed. Stirring of the solution has ciest and after 1 min of incubation, 100 µL of Tf 100 µM in PBS, pH 7.2 was added to the solution. Solutions were incubated for 30 min at 5 °C and transferred to dialysis tubing for 24 h dialysis in a 1 L PBS solution. Dialysis tubes were purchased from Spectrum labs, cat. No. 3787-D20; type: RC; MWCO: 12-14000. Dialysis tubing was treated vigorously with EDTA: tubing was immersed in 1 L of 2% sodium bicarbonate/1 mM EDTA solution in a 2 L glass beaker. Tubing was rinsed thoroughly with ddH2O (sterile ultra-pure water) and submerged completely in 50% ethanol/1 mM EDTA and stored at 4 °C. Tubing was rinsed thoroughly before use.
